# Mechanisms of Brain Aging and Their Links to Alzheimer’s and Parkinson’s Disease Pathology

**DOI:** 10.3390/ijms27188426

**Published:** 2026-09-21

**Authors:** Haiyi Yi, Haimeng Qin, Jo Aan Goon, Suzana Makpol, Jen Kit Tan

**Affiliations:** Department of Biochemistry, Faculty of Medicine, Universiti Kebangsaan Malaysia, Kuala Lumpur 56000, Malaysia

**Keywords:** brain aging, Alzheimer’s disease, Parkinson’s disease, neurodegeneration, cellular mechanisms, aging pathways

## Abstract

Brain aging is a major risk factor for neurodegenerative disorders and is associated with a progressive loss of neuronal homeostasis. Rather than resulting from a single pathological event, brain aging involves coordinated changes across multiple biological processes, including neuroinflammation, proteostasis impairment, mitochondrial dysfunction, neurotransmission deficit, cellular senescence, gut microbiota dysbiosis, extracellular vesicle dysregulation, nutrient-sensing disturbances, and metabolic drift. These processes interact across molecular, cellular, and systemic levels and may progressively increase neuronal vulnerability to neurodegenerative pathology. Age-related biological alterations may increase susceptibility to Alzheimer’s disease and Parkinson’s disease. Disease-associated proteins, including amyloid-β, tau, and α-synuclein, may interact with age-related inflammation, metabolic dysfunction, and loss of proteostasis. This review examines the evidence supporting these mechanisms, their interactions, and their relevance to normal brain aging, Alzheimer’s disease, and Parkinson’s disease. Because much of the current mechanistic evidence derives from animal and cellular models, further longitudinal and human studies are needed to provide a more complete understanding of how these mechanisms operate during human brain aging and neurodegenerative diseases.

## 1. Introduction

As global life expectancy increases, age-related neurological disorders are becoming an important public health concern. Aging is the main risk factor for neurodegenerative diseases, particularly Alzheimer’s disease (AD) and Parkinson’s disease (PD), which affect cognitive and motor function, synaptic plasticity, and neural network activity. Changes in synaptic and neural-network function may occur before clear neurodegeneration, indicating that biological changes during aging may increase vulnerability to these diseases [1]. These changes involve several cellular systems rather than a single mechanism. Earlier studies often examined mechanisms such as oxidative stress, mitochondrial dysfunction, impaired proteostasis, and neuroinflammation separately. Growing evidence, however, shows that these processes are closely connected. Dysfunction in one pathway may disrupt others, creating feedback loops that weaken neuronal homeostasis. Over time, molecular-level changes can lead to cellular and neural circuit dysfunction and contribute to cognitive decline [2].

Several biological processes are important components of this aging network. Chronic neuroinflammation [3], oxidative stress [4], impaired proteostasis [5], and mitochondrial dysfunction [6] contribute to the loss of neuronal resilience. Changes in neurotransmitter signaling [7] and cellular senescence [8] may further impair synaptic and neural network function. Emerging evidence shows that age-related alterations in the gut microbiota [9], extracellular vesicle (EV)-mediated communication [10], nutrient-sensing pathways [11], and metabolic regulation [12] may also affect the brain through systemic and cellular mechanisms. Several processes involved in normal brain aging may also interact with AD- and PD-specific pathology. Amyloid-β (Aβ), tau, and α-synuclein may amplify inflammatory, metabolic, mitochondrial, and proteostatic disturbances, thereby increasing neuronal vulnerability while contributing to disease-specific neurodegeneration.

**Figure 1:**
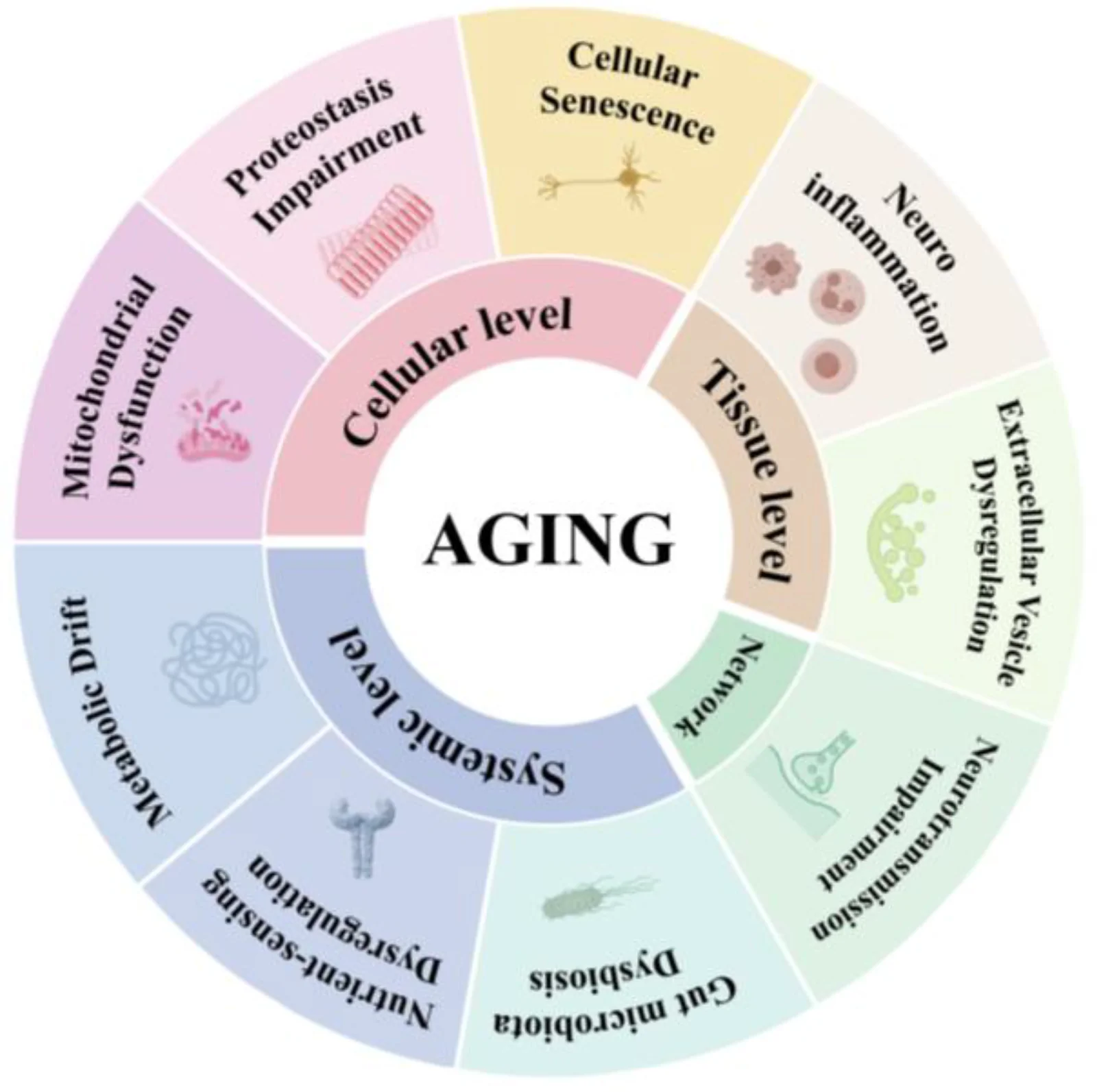
Conceptual overview of the nine mechanisms associated with brain aging. The mechanisms discussed in this review include neuroinflammation, impaired proteostasis, mitochondrial dysfunction, neurotransmission dysfunction, cellular senescence, gut microbiota dysbiosis, extracellular vesicle dysregulation, nutrient-sensing dysregulation, and metabolic drift. These interconnected processes may influence neuronal homeostasis and vulnerability to neurodegenerative pathology. Created using Figdraw.com (License ID: PUIYS6a7d3).

This review discusses the major molecular and cellular mechanisms involved in brain aging, their interactions, and how AD- and PD-related pathology may further aggravate these changes. Building on previous work examining individual processes, this review organizes the available evidence into nine interconnected pathways and compares their alterations during normal brain aging with those observed in AD and PD. Pathway-specific schematic figures summarize the major molecular and cellular interactions within each pathway, while Figure 1 provides an overview of the nine mechanisms discussed in this review.

## 2. Literature Search and Selection of Mechanistic Framework

This review applied an iterative, targeted literature search strategy. PubMed was used as the primary database, with supplementary searches conducted in Web of Science Core Collection and Scopus. In the first stage, broad brain-aging terms (“brain aging” OR “brain ageing” OR “neural aging” OR “neuronal aging”) were used to identify the major biological processes associated with brain aging and to establish the mechanistic framework of the review. Based on this initial screening, nine mechanisms were selected: neuroinflammation, proteostasis impairment, mitochondrial dysfunction, neurotransmission impairment, cellular senescence, gut microbiota dysbiosis, extracellular-vesicle dysregulation, nutrient-sensing dysregulation, and metabolic drift.

In the second stage, targeted searches were conducted for each mechanism. For each mechanism, three separate searches were conducted by pairing the mechanism-specific terms listed in Supplementary Table S1 with (1) brain-aging terms (“brain aging” OR “brain ageing” OR “neural aging” OR “neuronal aging”); (2) Alzheimer’s disease terms (“Alzheimer’s disease” OR AD); and (3) Parkinson’s disease terms (“Parkinson’s disease” OR PD). The search strategy was refined iteratively during manuscript development as additional pathway-, molecule-, and model-specific terms became relevant. Because this review was designed as a mechanistic narrative synthesis rather than a systematic review or meta-analysis, the search process was not restricted to a single fixed query.

Literature searches were conducted between October 2025 and 22 August 2026. Only English-language publications were considered, and no publication date restrictions were imposed. Studies were screened based on title and abstract relevance, followed by full-text assessment of studies addressing molecular, cellular, or systemic mechanisms involved in normal brain aging. Studies investigating AD and PD were considered when they provided relevant insights into how disease-associated pathology interacts with age-related biological alterations. As this was a narrative mechanistic review, a formal Preferred Reporting Items for Systematic Reviews and Meta-Analyses (PRISMA)-based quantitative screening process was not applied. Instead, studies were selected through relevance-based screening and evidence synthesis.

The nine mechanisms were selected primarily based on their representativeness in brain aging research, their association with age-related biological alterations and neurodegenerative pathology, and the potential interactions among different mechanisms. Review articles were used primarily to establish the research context, summarize field consensus, define the mechanistic framework, and identify relevant primary studies. Mechanistic statements were supported preferentially by primary research studies. Non-peer-reviewed preprints were not included.

The mechanistic pathways presented in this review were synthesized from complementary findings across human studies, animal models, and cell-culture systems. Because the complete sequence of each pathway has rarely been demonstrated within a single experimental model, the integrated pathways and schematic figures should be interpreted as conceptual syntheses rather than as fully established causal sequences. Model-specific information is provided in Supplementary Table S2, and proposed or incompletely validated relationships are described using cautious terminology. Longitudinal and human studies are required to validate the temporal and causal relationships among these mechanisms.

## 3. Neuroinflammation

Neuroinflammatory alterations are a common feature of brain aging and are characterized by a shift from tightly controlled immune surveillance toward an increasingly dysregulated immune state. The failure of inflammatory resolution results in sustained cytokine production, leading to a chronic, low-grade inflammatory state (inflammaging) that can occur without overt infection and may progressively disrupt neural function [13].

During normal brain aging, neuroinflammatory changes involve coordinated alterations in microglia, astrocytes, and inflammatory signaling pathways. Microglia are the brain’s primary innate immune cells. They are responsible for immune surveillance, phagocytic clearance of debris and pathogens, and the regulation of inflammatory responses. Microglia also contribute to neural circuit integrity by pruning synapses and supporting neuronal homeostasis [14,15]. Experimental studies, particularly in aged rodent models, describe microglial priming as a sensitized state, which is characterized by an exaggerated response to subsequent inflammatory stimulation [16]. In aged mice, genetic ablation of nucleotide-binding oligomerization domain-like receptor family pyrin domain-containing 3 (*Nlrp3*) reduced age-associated innate immune activation, central nervous system (CNS) transcriptional alterations, and astrogliosis and improved aspects of cognitive and motor function [17]. Damage-associated molecular patterns (DAMPs) released from stressed, damaged, or dying cells can engage pattern-recognition receptors and activate inflammatory signaling pathways [18]. These pathways can activate the inhibitor of κB (IκB) kinase (IKK) complex, which phosphorylates IκB and promotes its proteasomal degradation. This allows nuclear factor kappa B (NF-κB) to translocate to the nucleus. NF-κB signaling promotes the transcription of NLRP3 and pro-interleukin-1 beta (pro-IL-1β), as well as inflammatory mediators such as interleukin-6 (IL-6), tumor necrosis factor alpha (TNF-α), and various chemokines. Persistent NF-κB signaling and impaired inflammatory resolution may contribute to chronic low-grade inflammation during brain aging [19,20].

A number of experimental studies have identified that the NLRP3 inflammasome can be activated by diverse signals, including P2X purinoceptor 7 (P2X7)-mediated K^+^ efflux triggered by extracellular adenosine triphosphate (ATP), mitochondrial stress with mitochondrial reactive oxygen species (mtROS) and oxidized mitochondrial DNA (mtDNA) release, and lysosomal damage from protein aggregate uptake. These stimuli promote NLRP3-apoptosis-associated speck-like protein containing a caspase recruitment domain (ASC)-pro-caspase-1 complex assembly, which leads to caspase-1-dependent interleukin-1 beta (IL-1β)/interleukin-18 (IL-18) secretion [21]. In aged mice, reduced nicotinamide adenine dinucleotide phosphate (NADPH) oxidase 2 (NOX2)-derived reactive oxygen species (ROS) promoted microglial proliferation and IL-1β production through phagocyte oxidase 47-kDa subunit (p47phox)/extracellular signal-regulated kinase 1/2 (ERK1/2) signaling and were accompanied by increased protein tyrosine nitration. Similar age-related oxidative and inflammatory changes were observed in human post-mortem midbrain tissue, although these findings were observational [22]. In mice aged 17–24 months, complement component 1q (C1q) accumulation increased near synapses, although C1q deficiency did not lead to detectable synapse loss, and human hippocampal tissue likewise showed increased synaptic C1q [23]. In rhesus monkeys aged 21–30 years, increased synaptic C1q and reduced cluster of differentiation 47 (CD47) were associated with cognitive impairment. However, the study did not establish whether these changes contributed to synapse loss [24].

Under physiological conditions, astrocytes supply essential metabolic fuel to neurons, regulate extracellular neurotransmitter homeostasis, and help maintain the structural and functional integrity of the blood-brain barrier (BBB). Cell-culture and rodent studies have found that aging is accompanied by functional and transcriptional remodeling of astrocytes, including shifts toward reactive states and altered expression of inflammatory and oxidative mediators [25]. In mouse experimental systems, microglia-derived interleukin-1 alpha (IL-1α), TNF-α, and C1q induced a neurotoxic reactive astrocyte phenotype [26]. The relevance and heterogeneity of this phenotype in the normally aging human brain remain unclear. Experimental AD models indicate altered production of IL-1β, TNF-α, IL-6, and complement component 3 (C3) in reactive astrocytes, while human AD tissue also exhibits reactive-astrocyte and inflammatory alterations [27]. These changes may affect microglial activation and inflammasome-related signaling, but their effects differ across experimental and disease contexts.

In AD and PD, disease-associated proteins can further engage microglial pattern-recognition pathways and amplify inflammatory signaling. In AD-related experimental models, fibrillar Aβ activated cluster of differentiation 14 (CD14), Toll-like receptor 2 (TLR2), and Toll-like receptor 4 (TLR4)-dependent responses in neonatal primary microglia from wild-type and receptor-deficient mice [28]. Soluble Aβ42 oligomers also triggered TLR4-dependent inflammatory and neurophysiological responses in BV2 microglia, primary rat astrocytes, rat neuron–astrocyte co-cultures, and acute hippocampal slices from young rats [29]. In PD-related experimental models, conditioned medium containing α-synuclein from differentiated SH-SY5Y neuronal cells activated TLR2-dependent inflammatory responses in primary rat microglia. Similar TLR2-dependent responses were also observed in BV2 cells, primary microglia from wild-type and *Tlr2*-deficient mice, and a mouse model overexpressing α-synuclein [30]. α-Synuclein fibrils also interacted directly with the receptor for advanced glycation end products (RAGE) and induced RAGE-dependent inflammatory responses in primary mouse microglia, BV2 cells, receptor-modified cell systems, and biophysical binding assays [31]. In these experimental systems, activation of these receptors led to inflammatory signaling, although the downstream responses differed between ligands and models [28,29,30,31]. Taken together, these studies suggest that Aβ and α-synuclein partially overlap in their engagement of microglial inflammatory pathways in experimental AD- and PD-related systems. Importantly, neuroinflammation may also feed back to aggravate disease-associated pathology. Peripheral lipopolysaccharide (LPS) exposure promoted NLRP3-dependent microglial training, impaired Aβ clearance, and aggravated Aβ accumulation and cognitive deficits in a streptozotocin-induced mouse model of sporadic AD [32]. A study using J20 and amyloid precursor protein/presenilin 1 (APP/PS1) mouse models, together with complementary Aβ-injection experiments, provided evidence that C1q, C3, and microglial complement receptor 3 (CR3) contributed to early synaptic elimination [33]. Whether the same causal pathway operates to an equivalent extent in human AD remains uncertain. In male C57BL/6 mice, intrastriatal α-synuclein preformed fibril (PFF) injection with LPS increased microglial reactivity, α-synuclein pathology, and dopaminergic neuron loss, whereas partial microglial depletion with the colony-stimulating factor 1 receptor (CSF1R) inhibitor PLX5622 reduced these pathological changes [34]. These findings support a contribution of microglial activation to α-synuclein pathology in this experimental PD model, but their relevance to human PD remains to be established.

In the aging brain, neuroinflammation is associated with cellular damage, mitochondrial dysfunction, and oxidative stress. Cellular senescence, blood-brain barrier disruption, and impaired autophagy may further promote inflammation. Changes in the gut microbiota and metabolism may also contribute. Recent reviews of preclinical studies suggest that modulating inflammatory signaling in microglia and restoring their homeostatic functions may warrant further investigation in AD and PD; however, their clinical relevance remains unclear [35,36]. These related processes are discussed in the following sections. The main inflammatory pathways and the microglia–astrocyte feedforward loop are summarized in Figure S1.

## 4. Proteostasis Impairment

Proteostasis is the process by which cells maintain protein quality and prevent the accumulation of damaged or misfolded proteins. Evidence from animal and cellular studies suggests that proteostasis capacity may become less effective during brain aging. Under physiological conditions, proteostasis is maintained through the coordinated activity of the autophagy-lysosomal pathway, the ubiquitin-proteasome system (UPS), and molecular chaperone networks [37]. In mouse embryonic fibroblasts and human embryonic kidney 293 (HEK293) cells under nutrient- or energy-deprivation conditions, adenosine monophosphate (AMP)-activated protein kinase (AMPK)- and mechanistic target of rapamycin complex 1 (mTORC1)-dependent regulation of Unc-51-like kinase 1 (ULK1) and ULK1-mediated activation of the Beclin-1/vacuolar protein sorting 34 (VPS34) complex controlled autophagy initiation [38,39]. These experiments demonstrate a conserved cellular pathway, although they do not directly show whether it is altered with age in the human brain.

During aging, proteostasis regulation becomes progressively dysregulated, contributing to a vulnerable cellular state. Proteasome-activity assays and lysine 48 (K48)-polyubiquitin proteomics in the hippocampus and retrosplenial cortex of male and female rats showed age-, sex-, region-, and learning-dependent changes in UPS function, suggesting that proteasomal regulation does not decline uniformly across conditions. The UPS also targets proteins marked by K48-linked polyubiquitin chains for degradation by the 26S proteasome [40]. In the hippocampus of male C57BL/6J mice aged 3, 12, and 20 months, autophagic degradation and mechanistic target of rapamycin (mTOR)-related signaling changed with age and were associated with cognitive performance, while caloric restriction altered several of these outcomes [41]. Proteasome-associated deubiquitinases (DUBs) regulate ubiquitin-chain processing during 26S proteasome-mediated degradation [42]. Activity-based proteomics of aged mouse and killifish brains showed reduced activity of several redox-sensitive DUBs, whereas DUB inhibition in human induced pluripotent stem cell (iPSC)-derived neurons reproduced some age-associated changes in ubiquitination [43]. Molecular chaperones also contribute to the clearance of damaged proteins. In mouse HT22 hippocampal neuronal cells exposed to oxidative stress, 18-month-old SV129 mouse-brain homogenates, and complementary biochemical assays, heat shock protein 70 (HSP70) interacted with oxidatively damaged proteins and promoted their degradation by the 20S proteasome [44]. Neurons transdifferentiated from fibroblasts of older human donors showed changes in RNA-binding proteins, stress-granule responses, and heat shock protein 90 alpha (HSP90α) chaperone activity; selected findings were also examined in aged human and mouse brain tissue [45]. Together, these findings indicate model-dependent age-associated alterations in protein sorting, chaperone activity, and degradation capacity.

In aging *Drosophila*, impaired assembly of the 19S regulatory particle was associated with reduced 26S proteasome activity, whereas regulatory particle non-ATPase 11 (Rpn11) overexpression partly restored proteasome activity and suppressed age-related phenotypes [46]. In primary cortical neurons from embryonic Sprague-Dawley rats, exposure to 5 µM 4-hydroxynonenal impaired lysosomal acidification and hydrolase activity and reduced autophagic flux [47]. Because the latter findings were obtained using an acute cultured-neuron injury model, they do not provide direct evidence of lysosomal dysfunction during normal brain aging.

In replicatively aged *Saccharomyces cerevisiae* yeast mother cells, heat shock protein 104 (HSP104) overexpression enhanced protein disaggregation and restored UPS-substrate clearance [48]. Meanwhile, mathematical modeling suggested that oxidatively damaged proteins may become trapped in futile folding cycles with chaperones, reducing chaperone availability for healthy proteins and increasing protein misfolding and aggregation. These predictions were consistent with lifespan data from *Caenorhabditis elegans* [49]. Together, these findings suggest a proteotoxic feedback loop, although the full process has yet to be demonstrated in the aging human brain.

In experimental systems modeling protein-aggregation diseases, disease-associated proteins have been reported to affect different components of the proteostasis network. In biochemical assays, soluble Aβ oligomers inhibited mammalian 20S and 26S proteasomes by interfering with substrate-gate opening. Similar effects were observed for oligomeric α-synuclein and huntingtin in proteasome and brain lysate assays [50]. In a seeded human HEK293 tau-aggregation model, tau aggregates sequestered heat shock cognate protein 70 (HSC70)/HSP70, heat shock protein 90 (HSP90), and associated co-chaperones and impaired chaperone-dependent protein folding and vesicular trafficking [51]. In human postmortem PD brain samples, lysosome-associated membrane protein 2A (LAMP-2A) and HSC70 levels were reduced and regulatory microRNAs were altered in the substantia nigra pars compacta and amygdala. Consistent with these findings, experiments in SH-SY5Y cells showed that selected microRNAs reduced LAMP-2A or HSC70 expression and increased α-synuclein accumulation [52]. These model-specific findings suggest that AD- and PD-associated proteins can interact with proteostasis pathways, but they do not establish that either disease represents a continuation of normal aging.

Findings across these experimental systems suggest that age-related changes in protein degradation and chaperone function may reduce cellular and neuronal resilience, while AD- and PD-specific protein pathologies can impose additional proteostatic stress. Strategies targeting autophagy and proteostasis are still in the preclinical stage and require validation through appropriate human studies [53]. The proteostasis pathways discussed in this section are summarized in Figure S2.

## 5. Mitochondrial Dysfunction

Mitochondrial and redox alterations occur during brain aging, but their extent varies across species, brain regions, cell types, mitochondrial compartments, and experimental methods [54,55]. Under physiological conditions, mitochondria generate ATP through oxidative phosphorylation and contribute to calcium regulation and cellular redox homeostasis. Experimental aging studies identified changes in respiratory-chain activity, reactive oxygen species production, mitochondrial quality control, and antioxidant defenses. However, these findings should not be interpreted as a uniform sequence occurring across all aging brains.

In aging *Drosophila*, reduced complex I–III_2_ supercomplex assembly favored reverse electron transport (RET) at complex I and increased RET-derived ROS production, while RET inhibition reduced the age-related rise in ROS [56]. Whether a similar RET mechanism occurs in the aging human brain remains unclear. In cortical neurons isolated from 9- and 24-month-old rats, neurons from older animals showed reduced complex I substrate availability and lower complex IV reserve capacity [57]. Similarly, brain mitochondria isolated from 24-month-old rats exhibited reduced complex I activity and state 3 respiration, along with increased hydrogen peroxide production and cardiolipin peroxidation [58]. Neurologically normal human postmortem samples aged 42–97 years revealed a positive association between age and oxidative mitochondrial DNA damage in the cerebral cortex and cerebellum [59], although this association does not establish a causal link with functional decline. Additional experiments in aged mice reported alterations in oxidative phosphorylation complex activity [60], while a study using calcium-independent phospholipase A2 beta (iPLA2β)-deficient mice and primary neurons, together with viral rescue experiments, found that iPLA2β-dependent mitophagy was associated with cognitive impairment and inflammation [61].

In a long-term systemic lipopolysaccharide mouse model, age and NOX2 status affected microglial activation, oxidative stress, and dopaminergic neurodegeneration, with NOX2 deletion attenuating these effects [62]. However, this experimentally induced inflammatory model does not directly represent normal brain aging. In male rats aged 4, 14, and 24 months, monoamine oxidase activity and oxidative damage varied with age and brain region [63]. Histochemical analysis of adult female rat brains revealed cell- and region-specific iron distributions and suggested age-related changes in ferrous iron accumulation [64]. Age-dependent changes in antioxidant enzyme activity and glutathione levels were found across rat brain regions [65,66], while aged female C57BL/6J mice exhibited altered hippocampal nuclear factor erythroid 2-related factor 2 (NRF2)/ Kelch-like ECH-associated protein 1 (KEAP1) antioxidant responses and increased lipoxidative damage [67].

In mitochondria isolated from aged rat brains, cardiolipin peroxidation or depletion was linked to membrane depolarization and impaired respiratory function [56,68]. In aging mice, cyclophilin-D deficiency mitigated age-related dysfunction of mitochondrial F_1_F_o_ ATP synthase [69]. Evidence obtained across different experimental systems led to the proposal of a broader feedback relationship involving ROS production, mitochondrial permeability transition, and mitochondrial dysfunction [70], but this sequence has not been demonstrated in a single aging-brain model. In primary human fibroblasts, reduced basal mitophagy promoted senescence-associated phenotypes, whereas enhanced sequestosome 1 (p62)-mediated mitochondrial targeting partly reversed these effects [71], but whether these findings apply to aging human neurons remains unclear.

Aged Fischer 344 rats exhibited altered mitochondrial calcium buffering in acutely isolated basal forebrain neurons compared with young rats [72]. Transgenic mice older than 18 months had presynaptic calcium dysregulation alongside impaired hippocampal long-term potentiation (LTP) and cognitive function [73]. Age- and compartment-dependent changes in mitochondrial transport and cellular homeostasis were also observed in mouse peripheral neurons [74], but these findings may not extend to central neurons. In mice aged 3–18 months, age-related differences in hippocampal synaptic mitochondria were linked to emerging synaptic and memory impairment [75], while another study in aged female C57BL/6J mice found abnormalities in hippocampal mitochondrial bioenergetics, redox regulation, and calcium homeostasis [76]. In addition, evidence from experimentally induced injury models should be distinguished from normal aging. For example, NRF2-dependent responses were examined in aged mice subjected to focal cerebral ischemia [77], while blood-brain barrier alterations were studied in aged mice expressing endothelial NADPH oxidase 5 (NOX5) [78]. These findings reflect age-dependent responses under specific experimental conditions rather than general mechanisms of normal brain aging.

In AD-related human postmortem and experimental systems, mitochondrial abnormalities have been associated with Aβ and tau pathology. Biochemical experiments using mitochondria isolated from cerebellar granule cells found that Aβ exposure inhibited mitochondrial respiratory-chain activity, while synaptic-enriched human AD brain samples had similar alterations in respiratory-chain complexes [79]. In mixed rat or mouse astrocyte-neuron cultures, Aβ exposure activated astrocytic NADPH oxidase and increased ROS production, followed by mitochondrial membrane depolarization, glutathione depletion, and neuronal injury [80,81]. Tau accumulation at synaptic mitochondrial outer membranes was accompanied by impaired mitochondrial respiration in transgenic mice expressing human tau [82]. Proteomic and functional analyses of P301L-tau transgenic mice identified mitochondrial complex I and V abnormalities, along with reduced ATP production and increased oxidative stress [83]. Collectively, these studies suggest that Aβ and tau interact with mitochondrial and redox pathways in AD-related experimental systems. However, they were obtained from different human postmortem, biochemical, cell-culture, and transgenic-mouse studies and therefore do not establish a single causal sequence in human AD.

PD-related experimental models have revealed several links between mitochondrial stress and dopaminergic-neuron vulnerability. Astrocyte-specific, inducible monoamine oxidase B (MAO-B) overexpression in transgenic mice increased MAO-B activity and hydrogen peroxide production, accompanied by neuroinflammation, impaired mitochondrial complex I activity, and loss of nigral dopaminergic neurons [84]. Glutathione metabolism was also disrupted in cultured rat N27 dopaminergic neuronal cells expressing A53T α-synuclein under proteasome inhibition, associated with reduced glutathione levels and increased cellular stress [85]. However, this combined genetic and pharmacological cell-culture model does not reproduce the full pathological environment of human PD. Its relevance should be interpreted cautiously. Age-dependent effects of parkin deficiency were observed in clustered regularly interspaced short palindromic repeats (CRISPR)-generated parkin-deficient monkeys, with substantia nigra neurodegeneration and increased phosphorylated α-synuclein accumulation occurring in adult but not younger animals [86]. In MitoPark mice, the conditional disruption of mitochondrial transcription factor A in dopaminergic neurons impaired respiratory chain function. Meanwhile, substantia nigra pars compacta neurons were more vulnerable to redox stress and calcium load than other populations of dopaminergic neurons [87]. These models link α-synuclein accumulation, parkin deficiency, astrocytic MAO-B activity, proteostatic stress, and calcium load to mitochondrial dysfunction in PD, but do not establish PD as a direct extension of normal brain aging.

Human postmortem studies and experimental models in rodents, *Drosophila*, nonhuman primates, and cultured cells suggest that mitochondrial and redox features of aging vary across models and experimental systems. In AD and PD, disease-specific genetic and proteinopathic processes may further interact with age-related mitochondrial vulnerability. Approaches targeting mitophagy and mitochondrial quality control are still largely preclinical and need further evaluation in human studies [88]. These mitochondrial and redox alterations are summarized conceptually in Figure S3.

## 6. Neurotransmitter Transmission Impairment

During the aging process of the brain, dysfunction in neurotransmitter transmission is mainly manifested as a gradual decline in synaptic communication capacity and plasticity across multiple neurotransmitter systems. Rather than resulting from a simple reduction in individual neurotransmitters, aging-related synaptic dysfunction involves interrelated changes across multiple aspects, including neurotrophic support, glutamatergic plasticity, cholinergic signaling, and dopaminergic modulation, which ultimately influence synaptic integration and neural circuit function [89].

Age-related cholinergic dysfunction involves alterations in neurotrophic signaling, acetylcholine-related function, and postsynaptic responsiveness. Cultured basal forebrain cholinergic neurons (BFCNs) derived from young and aged Sprague-Dawley rats showed reduced tropomyosin receptor kinase A (TrkA)-mediated retrograde pro-nerve growth factor (proNGF) transport and increased p75 neurotrophin receptor (p75NTR)-mediated transport in aged neurons. These alterations were accompanied by increased nitrosative stress [90]. In the cortex and subcortical structures of 2- and 24-month-old male Sprague-Dawley rats, high-affinity choline uptake, choline acetyltransferase (ChAT) activity, and muscarinic acetylcholine receptor density were all reduced with age, consistent with diminished cholinergic function [91]. This reduction in cholinergic responsiveness was also evident functionally. Electrophysiological recordings of CA1 pyramidal neurons in hippocampal slices from Fischer 344 and Sprague-Dawley rats of different ages revealed an age-related reduction in cholinergic slow excitatory postsynaptic potential (EPSP) amplitude [92]. Age-related cholinergic dysfunction therefore involves neurotrophic signaling, acetylcholine-related function, and postsynaptic responsiveness. However, this evidence comes mainly from cultured neurons and rodent brain tissue, and whether the same processes occur in the aging human brain remains unclear.

Age-related changes in the glutamatergic signaling also affect synaptic function. In the hippocampal CA1 region of 16–24-month-old male Fischer 344 rats, reduced expression of the N-methyl-D-aspartate (NMDA) receptor subunit 2B (NR2B) impaired NMDA-dependent LTP induction and spatial learning. Experimental reduction in NR2B in young rats reproduced part of the age-related phenotype, whereas pharmacological enhancement of NR2B-related signaling improved LTP in aged animals, providing experimental support for a role of NR2B signaling in age-related deficits in synaptic plasticity and spatial learning [93]. A separate study of aged Sprague-Dawley rats aged 24–27 months found reduced hippocampal expression of the glutamate-aspartate transporter (GLAST) and glutamate transporter 1 (GLT-1), together with impaired glutamate uptake. In corresponding hippocampal slices, inhibition of glutamate uptake enhanced N-methyl-D-aspartate receptor (NMDAR)-dependent responses and produced more pronounced metabotropic glutamate receptor (mGluR)-dependent effects in aged tissue, accompanied by impaired long-term depression (LTD) in CA1 pyramidal neurons [94]. Thus, age-related disruption of glutamatergic homeostasis impacts both receptor function and glutamate clearance, which are related to impaired hippocampal synaptic plasticity. However, direct evidence of comparable glutamatergic dysfunction in normal human brain aging remains limited.

Aging-associated dopaminergic dysfunction involves alterations in dopamine transport, receptor signaling, and dopaminergic regulation of other neurotransmitter systems. Striatal synaptosomes obtained from 6-, 18-, and 24-month-old male Fischer 344 rats showed an age-related reduction in dopamine uptake accompanied by decreased plasma-membrane dopamine transporter (DAT) expression [95]. In striatal membrane preparations from 40-month-old female Wistar rats, both D1 and D2 receptor densities were reduced, with a particularly pronounced decrease in the high-affinity state of the D1 receptor, together with reduced dopamine-stimulated adenylyl cyclase activity [96]. These alterations extend beyond dopamine handling itself to the regulation of other neurotransmitter systems. In vivo microdialysis of the striatum in male Fischer-344 rats aged 6, 15, and 25 months showed that local quinpirole administration suppressed acetylcholine release, whereas this inhibitory response was attenuated in aged rats. The altered dose–response pattern across age groups indicated age-related changes in D2 receptor-mediated regulation of cholinergic transmission [97]. Human evidence provides a complementary systems-level perspective. Among individuals aged 20–79, lower D1 receptor availability, as measured by positron emission tomography (PET), was associated with reduced caudate-cortical functional organization differentiation and poorer memory performance [98]. Therefore, age-related dopaminergic changes affect neurotransmitter transport, receptor signaling, inter-system regulation, and corticostriatal network organization.

In neurodegenerative disease contexts, synaptic dysfunction is additionally shaped by disease-specific molecular pathologies that are distinct from normal aging-related changes. Amyloid precursor protein (APP)-related pathology links endosomal dysfunction to impaired cholinergic neurotrophic signaling. Basal forebrain cholinergic neurons from 12-month-old Ts65Dn mice and their 2N littermate controls had enlarged Rab5-positive early endosomes and increased Rab5 activation associated with *App* gene dosage. In PC12M cells and primary BFCNs derived from embryonic day 18 (E18) Sprague-Dawley rats, APP β-carboxyl-terminal fragment (β-CTF)/C99 increased Rab5 activation and endosomal enlargement and impaired retrograde nerve growth factor (NGF) transport, while Rab5 inhibition improved the neuronal phenotype. Rab5 inhibition also reduced APP-associated axonal transport blockages in a *Drosophila* APP695 transgenic model [99]. Aβ affects glutamatergic synaptic function. Soluble Aβ oligomers reduced NMDAR-mediated Ca^2+^ signaling in dendritic spines of CA1 neurons in rat organotypic hippocampal slices and gradually decreased dendritic spine density and excitatory synapse number. Targeting NMDAR, calcineurin, or cofilin prevented these effects, linking the NMDAR-calcineurin-cofilin pathway to Aβ oligomer-induced synaptic dysfunction in this ex vivo AD-related model [100]. α-Synuclein-related pathology can disrupt dopaminergic regulation through altered tyrosine hydroxylase (TH) phosphorylation and protein phosphatase 2A (PP2A) activity. In MN9D dopaminergic cells and wild-type α-synuclein (WT-Syn) and A53T-Syn transgenic mice, α-synuclein overexpression was associated with reduced TH phosphorylation and activity and increased PP2A activity, while α-synuclein knockout produced the opposite pattern. The dephosphorylation-mimetic S129A mutant more strongly inhibited TH and activated PP2A than WT α-synuclein in MN9D cells, an α-synuclein knockout (ASKO) mouse transduction model, and a recombinant protein system, whereas Ser129 phosphorylation attenuated these effects [101]. Given that TH is the rate-limiting enzyme in catecholamine synthesis, this mechanism links α-synuclein Ser129 phosphorylation to PP2A activity and TH-dependent regulation of dopamine synthesis.

Overall, neurotransmitter transmission impairment during brain aging represents a progressive disruption of synaptic communication, extending from reduced neurotransmitter signaling and impaired synaptic plasticity to broader dysfunction of neural circuit coordination. Experimental AD- and PD-related models suggest that APP/β-CTF, Aβ, and α-synuclein abnormalities are implicated in disturbances of cholinergic neurotrophic transport, glutamatergic synaptic homeostasis, and dopamine synthesis. However, most mechanistic evidence comes from animal, cellular, and ex vivo models, and its relevance to human AD and PD remains uncertain. Therapeutic strategies targeting synaptic function have advanced from preclinical studies to early clinical investigation, although their efficacy and safety require further evaluation [102]. Age-related changes in the major neurotransmitter systems are summarized in Figure S4.

## 7. Cellular Senescence

Cellular senescence in brain aging is characterized by a progressive transition toward a senescence-like state in response to persistent cellular stress and damage, which is accompanied by senescence-associated secretory phenotype (SASP). Because neural cell types differ in their proliferative capacity, the manifestations of cellular senescence may vary across the aging brain. In particular, although stable cell-cycle arrest is a canonical feature of cellular senescence, this criterion alone is insufficient for post-mitotic neurons. Neuronal senescence therefore requires assessment of multiple complementary senescence-associated features rather than reliance on a single marker [103]. This process represents a progressive senescence program in which persistent damage signaling is reinforced through interconnected transcriptional and secretory regulatory networks.

DNA damage response (DDR) signaling initiates stable cell-cycle arrest by activating the p53–p21 axis and the cyclin-dependent kinase inhibitor 2A (p16^INK4a^)–cyclin-dependent kinase 4/6 (CDK4/6)–retinoblastoma protein (RB) pathway, thereby maintaining RB in a hypophosphorylated state and suppressing E2F transcription factor-dependent transcription required for cell-cycle progression. In replicative and TNF-α-induced senescent human primary umbilical vein endothelial cells (HUVECs), p21 and p16 expression increased, accompanied by transcriptional repression of genes involved in cell-cycle regulation, DNA replication and repair, and chromatin organization. Many of these downregulated genes were targets of E2F and its dimerization partner, RB-like E2F and multi-vulval class B (DREAM), which is consistent with stable cell-cycle arrest during senescence [104]. DDR contributes to SASP-associated inflammatory signaling through GATA binding protein 4 (GATA4). Human fibroblast senescence models demonstrated that DDR stabilizes GATA4 by preventing its selective autophagic degradation via p62, which leads to NF-κB activation through TRAF3-interacting protein 2 (TRAF3IP2) and IL-1α, resulting in increased SASP production. GATA4 accumulation was also observed in the human prefrontal cortex with age, though this evidence was only correlative [105]. Together, these studies highlight stable cell-cycle arrest and DDR-dependent inflammatory signaling as distinct features of cellular senescence. However, most mechanistic evidence comes from proliferative cell models, while evidence from the aging human brain remains correlative.

SASP production and maintenance are regulated by multiple signaling pathways. H_2_O_2_-induced senescent NIH3T3 and MRC-5 fibroblasts showed early NF-κB activation and later signal transducer and activator of transcription 3 (STAT3)-dependent SASP maintenance through positive feedback [106]. Human fibroblast senescence models further linked the mTOR–mitogen-activated protein kinase-activated protein kinase 2 (MAPKAPK2) pathway to increased stability of SASP-associated messenger RNAs (mRNAs) and sustained SASP expression [107]. SASP factors can also transmit senescence to neighboring cells. Studies using co-culture, transwell, and conditioned-medium systems with human fibroblasts and mammary epithelial cells found stable paracrine senescence, with inflammasome-IL-1 signaling involved in SASP regulation [108]. These studies define general mechanisms of SASP regulation and paracrine senescence, but they were conducted mainly in non-neural cellular and tissue models.

Microglial SASP can directly affect neuronal synaptic function. In mice with inducible microglia-specific *Pfn1* deletion, microglial SASP, which is driven by extracellular signal-regulated kinase (ERK)/NF-κB signaling, can impair mitochondrial function at synapses and reduce gamma-aminobutyric acid (GABA)-ergic synaptic transmission in the prefrontal cortex, thereby contributing to circuit dysfunction and behavioral alterations [109]. Overall, these changes link microglial senescence-associated signaling to synaptic dysfunction and altered neural circuit function, highlighting a potential mechanism through which age-related changes in microglia may affect neuronal function.

In neurodegenerative diseases, age-related changes are further influenced by disease-specific molecular mechanisms. In AD, Aβ_1−42_ oligomer exposure in primary astrocytes from human AD donors was associated with calcium dyshomeostasis, mitochondrial dysfunction, DNA damage marked by phosphorylated histone H2AX (γH2AX), p14 alternate reading frame (p14^ARF^) upregulation, and SASP induction, consistent with the acquisition of a senescence-like phenotype in these cells [110]. In PD, adeno-associated virus (AAV)-mediated expression of nuclear-targeted α-synuclein in midbrain dopaminergic neurons of male C57BL/6J mice induced oxidative stress and DNA damage, accompanied by upregulation of p53 and p21 and a broad SASP-related inflammatory gene signature. These changes occurred along with glial activation and a pro-inflammatory milieu, which may contribute to neuronal dysfunction and dopaminergic neuron loss [111]. Despite the disparities in upstream triggers, these disease-associated senescence responses converge on enhanced inflammatory signaling, which may contribute to the progression of neurodegenerative pathology.

Therefore, cellular senescence is not a passive degenerative process but involves coordinated responses to persistent cellular stress and damage, together with SASP-mediated inflammatory signaling. This process involves interconnected changes in damage responses, stable cell-cycle arrest, secretory reprogramming, and altered neuronal function, and is further shaped by disease-specific pathological mechanisms under neurodegenerative conditions. These changes connect genomic instability and cellular inflammatory responses with impaired neuronal and neural circuit function, although the underlying mechanisms vary across cell types and experimental models. Therapeutic strategies targeting cellular senescence, including senolytics and senomorphics, have shown potential to alleviate senescence-associated inflammation and functional decline in preclinical models of brain aging and neurodegeneration [112]. The main pathways involved in cellular senescence in the aging and neurodegenerative brain are shown in Figure S5.

## 8. Gut Microbiota Dysbiosis

Gut microbiota dysbiosis during aging is characterized by alterations in microbial composition and function, including changes in the abundance of short-chain fatty acid (SCFA)-producing bacteria and reduced availability of beneficial microbial metabolites. Human cohort studies and experimental models have reported age-associated alterations in gut microbial composition and microbial metabolites. However, the direction of this relationship is unclear, as microbial remodeling may reflect age-related changes in the host environment or contribute to inflammatory and metabolic dysregulation [113].

Aging-related declines in SCFA-producing bacteria, particularly reduced butyrate availability, may contribute to immune-cell senescence by altering inflammatory and metabolic pathways. In a human cohort study comparing healthy young adults aged 18–37 years with older adults aged ≥60 years, older individuals exhibited lower fecal and serum butyrate levels, and reduced fecal butyrate levels were correlated with increased frequencies of cluster of differentiation 28 (CD28)-negative, cluster of differentiation 57 (CD57)-positive senescent cluster of differentiation 4 (CD4)-positive T cells (CD28^−^CD57^+^ CD4^+^ T cells). In an ex vivo senescent T-cell model derived from older donors and activated by cluster of differentiation 3 (CD3) stimulation, butyrate supplementation reduced senescence-associated features, including γH2AX and phosphorylated p53 (p-p53) expression, and suppressed NF-κB and mTORC1 signaling as well as mitochondrial ROS accumulation. These effects were accompanied by decreased secretion of SASP-related cytokines, including IL-6, interleukin-8 (IL-8), and IL-1β [114]. Dietary fiber intervention in aged male Balb/c mice increased gut-derived SCFA availability and reduced age-associated inflammatory responses. A high-fiber diet containing inulin elevated cecal butyrate and acetate levels, while decreasing inflammatory gene expression in microglia and hippocampal tissue, including *Il1b, Tnf, Il6, Nlrp3*, and *Tlr4*. Direct sodium butyrate administration further reduced LPS-induced inflammatory responses in aged microglia and hippocampus, supporting the involvement of gut-derived SCFAs in regulating neuroinflammatory states during aging [115]. Comparison of young and aged male C57BL/6 mice using microbiome sequencing and multi-tissue metabolomic profiling revealed age-related gut microbial remodeling and systemic accumulation of microbiota-derived metabolites, including trimethylamine N-oxide (TMAO) and indole-3-acetic acid (IAA). These metabolic alterations were accompanied by tissue-specific changes in lipid metabolism, redox regulation, and neurochemical pathways across multiple organs, including the cerebral cortex [116]. These studies indicate that age-related gut microbial changes coincide with alterations in immune and metabolic regulation and may influence neuroinflammatory states through peripheral inflammatory pathways.

In male C57BL/6 mice, reduced expression of tight junction proteins such as occludin and zonula occludens-1 (ZO-1) was associated with increased intestinal epithelial permeability, elevated circulating LPS levels, and activation of the TLR4/myeloid differentiation primary response 88 (MyD88) signaling pathway, accompanied by NF-κB-mediated systemic inflammatory responses and inducing IL-6 and TNF-α release. These circulating inflammatory signals may subsequently affect the central nervous system through compromised blood-brain barrier integrity, promoting gut-brain inflammatory communication [117]. Under these conditions, disruption of intestinal barrier integrity facilitates the propagation of systemic and neuroinflammatory responses.

In the central nervous system, microbial dysbiosis influences neuroinflammatory signaling through disrupted bile acid metabolism. In aged male C57BL/6 mice together with BV-2 microglia models, dysbiosis, including a reduction in bile salt hydrolase (BSH)-associated bacteria, was associated with increased accumulation of bile acid metabolites such as tauro-β-muricholic acid (TβMCA) in the brain, which promotes a pro-inflammatory microglial phenotype and activates NLRP3 inflammasome-associated inflammatory signaling in the hippocampus and hypothalamus [118]. Concurrently, single-cell transcriptomic analyses of hippocampal microglia from aging male C57BL/6 mice revealed that microglia progressively transitioned toward an activated phenotype characterized by increased expression of complement components C1q and C3, accompanied by upregulation of activation-associated genes such as C-type lectin domain family 7 member A (*Clec7a*), reflecting age-related remodeling toward a more inflammatory microglial state [119]. Age-related microglial activation is also linked to structural changes in neural tissue. Sustained microglial activation is associated with white matter structural alterations, reduced myelin integrity, and decreased presynaptic density, changes that correlate with impaired cognitive performance in aged C57BL/6J mice [120]. Together, alterations in gut-derived metabolites and age-related microglial state transitions provide potential links between peripheral microbial changes, neuroinflammatory activation, and cognitive vulnerability during aging.

In neurodegenerative disease contexts, gut microbiota-associated neuroinflammatory signaling is further shaped by disease-specific mechanisms. In AD, microbiota-derived SCFAs can modulate microglial activation and phagocytic function, thereby influencing Aβ deposition. In germ-free and specific pathogen-free APPPS1 mouse models, SCFA supplementation increased Aβ plaque deposition and altered microglial transcriptional profiles, including changes in apolipoprotein E (ApoE) expression, microglial recruitment, and phagocytic function, suggesting that microbiota-derived SCFAs influence AD pathology through microglial modulation [121]. Meanwhile, in APP23 transgenic mice with astrocyte-specific IκB kinase 2 (IKK2)/NF-κB activation, astrocytic NF-κB signaling induced distinct microglial responses, characterized by increased microglial reactivity and enhanced Aβ clearance capacity, resulting in reduced amyloid plaque burden. This illustrates the cell-type-dependent effects of inflammatory signaling in AD [122]. In C57BL/6 mice, intestinal injection of α-synuclein PFFs into the pylorus/duodenum of 3-month-old mice induced transneuronal propagation to the brain via the vagus nerve, which was blocked by vagotomy, supporting a causal role of gut-to-brain α-synuclein transmission in PD-like pathology [123]. In *Tlr4*-deficient C57BL/10ScNJ mice and wild-type controls receiving intrastriatal injection of human α-synuclein PFFs, loss of TLR4 signaling reduced microglial clearance of α-synuclein and was associated with enhanced α-synuclein propagation and dopaminergic neuronal damage in the substantia nigra [124].

Dietary factors also interact with gut microbiota, inflammatory responses, and brain aging. Omega-3 polyunsaturated fatty acids (n-3 PUFAs), particularly eicosapentaenoic acid (EPA) and docosahexaenoic acid (DHA), participate in inflammatory regulation through the production of specialized pro-resolving mediators, which promote the resolution of neuroinflammation and influence microglial activity [125]. n-3 PUFAs can also affect gut microbial composition and metabolite production, with reported effects on intestinal barrier integrity and systemic inflammation [126]. In older adults, randomized controlled trials of microbiota-directed interventions, including probiotics, prebiotics, and synbiotics, have reported changes in specific microbial populations and inflammatory markers, indicating their potential relevance for age-related gut dysbiosis [127]. Overall, gut microbiota dysbiosis integrates metabolic alterations, intestinal barrier disruption, and immune activation, providing a potential route through which peripheral microbial and metabolic changes may influence central neuroinflammation and neural function. In neurodegenerative diseases, these processes are further amplified by disease-specific pathological mechanisms, thereby potentially increasing neuroinflammatory signaling and neuronal vulnerability. The links between gut dysbiosis, inflammation, and neurodegeneration are summarized in Figure S6.

## 9. Extracellular Vesicle Dysregulation

Extracellular vesicle dysregulation in brain aging is not merely a quantitative alteration in vesicle secretion but reflects coordinated changes in EV biogenesis, molecular cargo composition, and intercellular communication. These alterations are closely associated with dysfunction of the endosomal pathway and cellular senescence, leading to dysregulated intercellular communication and inflammatory signaling. Aging-related EV remodeling involves changes in vesicle production, cargo composition, recipient-cell uptake, and extracellular processing, which potentially sustain inflammatory signaling. Therefore, EV dysregulation represents an important interface linking peripheral and central inflammatory processes during brain aging.

Remodeling of EV biogenesis represents an early feature of EV dysregulation during brain aging. In an in vitro model of aged rat cortical neurons, aging neurons exhibited enhanced EV release through the endosome-multivesicular body (MVB) pathway together with increased heterogeneity in vesicle number and size distribution [128]. In parallel, circulating EVs from aged individuals and aged mice showed significant alterations in quantity, size, and immunomodulatory function, although these effects appeared to depend on cellular origin and biological context [129]. Telomere stress and attrition further influence EV biogenesis and cargo composition. In transcription elongation factor A (SII) 1-knockout (*Tcea1^−^/^−^*) mouse embryonic fibroblasts (MEFs) and aged primary cells, transcription factor IIS (TFIIS) loss triggered R-loop-dependent release of telomeric DNA into EVs [130], while studies in telomerase reverse transcriptase-knockout (*Tert^−^/^−^*) mice and human plasma samples revealed that telomere shortening altered EV abundance and functional properties [131]. These studies indicate that age-related cellular stress can reshape EV biogenesis, although most mechanistic evidence currently comes from peripheral or non-neuronal models.

Beyond changes in EV production, aging also affects EV cargo composition, which can modify inflammatory responses in recipient cells. In a cellular senescence model, EVs derived from senescent cells are enriched in microRNA-30b-5p (miR-30b-5p), which directly targets and suppresses sirtuin 1 (SIRT1) expression. This relieves SIRT1-mediated inhibition of NF-κB signaling, promotes p65 nuclear translocation, and thereby drives transcriptional upregulation of IL-1β and IL-6 in recipient macrophages, ultimately promoting a pro-inflammatory phenotype [132]. Although this mechanism was demonstrated primarily in fibroblast-derived EVs and macrophage models rather than neural cell systems, it provides evidence that senescence-associated EV cargo can influence inflammatory transcriptional responses in recipient cells. Thus, EV-mediated signaling may participate in inflammatory communication between aging cells.

At the level of recipient cells, senescence-associated EV signaling can establish a feedback mechanism that sustains inflammatory communication. EVs derived from senescent cells induce macrophage activation and promote IL-6 expression through the miR-30b-5p/SIRT1/NF-κB pathway [132]. In addition, experimental studies in senescent macrophage models showed that senescent immune cells, including macrophages, can release EVs carrying pro-inflammatory molecular cargo that may further modify inflammatory responses in neighboring cells [133]. Dipeptidyl peptidase 4 (DPP4) enrichment on senescence-associated EVs suppresses cellular uptake by non-phagocytic cells, whereas macrophages can efficiently internalize these EVs through phagocytic pathways [134]. These mechanisms indicate that senescence-associated EVs can regulate inflammatory interactions between aging cells, but the extent to which these mechanisms operate in neural cell populations during brain aging remains to be established.

In neurodegenerative disease contexts, this axis is selectively amplified at critical nodes. In primary rat hippocampal neurons, exosomes mediated intercellular tau transfer and seeded tau aggregation in recipient cells, while tau-containing exosomes were also detected in cerebrospinal fluid from patients with AD, although the human samples did not directly demonstrate trans-synaptic propagation [135]. Meanwhile, plaque-associated microglial neurodegenerative phenotype (MGnD) in an amyloid mouse model exhibited increased expression of EV-associated proteins, including tumor susceptibility gene 101 (TSG101) and cluster of differentiation 9 (CD9), and released increased amounts of EVs enriched in phosphorylated tau, facilitating tau propagation across brain regions [136]. Pathogenic tau further activates microglial NF-κB signaling, which promotes tau release and spreading while enhancing neuroinflammatory responses, including IL-1β and TNF production. In PS19 tauopathy mice, selective manipulation of microglial NF-κB showed that activation enhanced tau seeding and spreading, whereas inactivation reduced tau propagation and ameliorated spatial learning and memory deficits [137]. Together, the available evidence supports a potential feed-forward interaction linking EV-mediated tau propagation, microglial inflammation, and tau pathology.

In PD, the effect of EVs on α-synuclein homeostasis depends on their molecular cargo. In PD patient serum-derived EVs and 1-methyl-4-phenyl-1,2,3,6-tetrahydropyridine (MPTP)-treated mouse models, EV-carried transfer RNA-derived fragment 02514 (tRF-02514) suppressed autophagy-related 5 (ATG5)-dependent autophagy and activated NLRP3/Caspase-1/gasdermin D (GSDMD)-mediated pyroptosis signaling in microglia, promoting IL-1β/IL-18 release and neuroinflammation, whereas tRF-02514 knockdown alleviated pyroptosis and protected dopaminergic neurons [138]. In contrast, using brain-derived EVs from α-synuclein knockout mice combined with α-synuclein PFF seeding models, studies showed that brain-derived EVs containing active cathepsins B and S promoted the proteolytic degradation of α-synuclein species, reduced their seeding capacity, and restricted pathological spread [139]. These findings suggest that EVs derived from different cellular sources and disease contexts can have distinct effects on pathological protein handling, either supporting the spread of pathogenic proteins or facilitating their extracellular clearance.

In conclusion, EV dysregulation represents a link between age-related cellular stress, altered intercellular communication, and neurodegenerative pathology. However, current evidence remains heterogeneous, with studies involving aged animals, cultured cells, peripheral EVs, and disease models. The contribution of EV-mediated mechanisms to normal human brain aging, as well as their distinction from disease-specific processes in AD and PD, remains to be further clarified. Therapeutic approaches targeting EV dysregulation, including inhibition of pathogenic EV release, administration of stem cell-derived EVs, and engineered EV-based delivery systems, have shown potential in modulating pathological protein propagation, neuroinflammatory responses, and neuronal function in preclinical models of AD, PD, and other neurodegenerative disorders [140]. Age-related changes in EV production, cargo, and clearance are summarized in Figure S7.

## 10. Nutrient-Sensing Dysregulation

Nutrient-sensing dysregulation during brain aging does not arise from alterations in a single signaling pathway, but rather reflects the coordinated disruption of major metabolic signaling networks, particularly the insulin-like growth factor 1 (IGF-1)/ phosphoinositide 3-kinase (PI3K)-protein kinase B (AKT) axis and the AMPK-mTORC1 axis. A hallmark of this process is the gradual loss of balance between anabolic and catabolic regulation, resulting in impaired metabolic homeostasis. These changes occur along a continuum involving weakened nutrient signaling, impaired energy sensing, autophagy impairment, and inflammatory activation. These pathways interact through shared downstream regulators involved in proteostasis, mitochondrial function, and inflammatory responses, thereby linking altered nutrient availability and energy status to cellular vulnerability during brain aging.

Attenuation of IGF-1/PI3K–AKT signaling represents an early feature of nutrient-sensing dysregulation during brain aging. In female BALB/c mice, age-associated alterations in hippocampal IGF-1 signaling and downstream PI3K–AKT activity have been reported, although the direction and magnitude of these changes appear to be tissue- and context-dependent [141]. In parallel, age-related upregulation of nuclear receptor subfamily 4 group A member 1 (NR4A1) has been shown to inhibit AKT signaling, thereby reducing inhibitory phosphorylation of glycogen synthase kinase 3 beta (GSK-3β) and promoting tau hyperphosphorylation in aging-related models. Additionally, impaired AKT signaling also compromises synaptic maintenance and neuronal resilience, increasing neuronal vulnerability to metabolic and oxidative stress [142].

At the energy-sensing level, dysregulation of the AMPK–mTORC1 axis is associated with impaired autophagic homeostasis during brain aging. In the cortex and hippocampus of Sprague-Dawley rats, altered AMPK/mTOR/ULK1 signaling was associated with reduced neuronal autophagy, whereas pharmacological activation of AMPK enhanced autophagic activity and improved age-related brain functional decline [143]. In C57BL/6J mice aged 19–21 months, dysregulation of mTORC1-related signaling and p70 ribosomal S6 kinase (p70S6K) activity altered translational regulation and was accompanied by impaired synaptic plasticity and cognitive decline [144]. Overall, age-related disruption of nutrient sensing may affect both autophagy and protein synthesis, thereby contributing to impaired proteostasis.

At the axis-integration level, the sirtuin family serves as a central coupling hub that links nutrient-sensing dysfunction to coordinated disturbances in inflammation, mitochondrial homeostasis, and genomic maintenance. Reduced SIRT1 expression relieves suppression of NF-κB signaling and promotes IL-1β-mediated neuroinflammation in a microglia-specific SIRT1 conditional knockout (*LysM-Cre/Sirt1^F/F^*) mouse model [145]. In addition, impaired SIRT1-mediated deacetylation increases peroxisome proliferator-activated receptor gamma coactivator 1-alpha (PGC-1α) acetylation, which is associated with reduced mitochondrial respiration, decreased ATP production, and accelerated cellular senescence in brain endothelial cells [146]. Similarly, age-related reduction in sirtuin 3 (SIRT3) expression has been associated with increased superoxide dismutase 2 (SOD2) acetylation, reduced antioxidant activity, and accumulation of oxidative stress in the auditory cortex [147]. Because this evidence was obtained specifically in the auditory cortex, it should not be generalized to the entire aging brain. In addition, in sirtuin 6 (*Sirt6*)-knockout mouse embryonic fibroblasts and aged mouse tissues, dissociation of SIRT6 from long interspersed nuclear element-1 (LINE-1) retrotransposon loci derepresses LINE-1 expression and promotes cytoplasmic accumulation of LINE-1 complementary DNA (cDNA), which activates DNA damage responses and cyclic GMP-AMP synthase (cGAS)-dependent type I interferon signaling, thereby contributing to age-associated genomic instability and inflammatory activation during aging [148,149]. Sirtuins may integrate nutrient-sensing signals with inflammatory, mitochondrial, and genomic homeostasis, acting as downstream effectors rather than independent nutrient-sensing pathways.

Nutritional factors may also influence nutrient-responsive pathways and downstream processes associated with brain aging. In female BALB/c mice subjected to alternate-day dietary restriction, dietary restriction modified IGF-1 signaling in an age- and tissue-dependent manner, indicating that nutritional availability can reshape nutrient-sensing responses during aging [141]. Omega-3 polyunsaturated fatty acids provide another nutritional example. In aged rats, dietary EPA reduced age-associated increases in IL-1β and p38 mitogen-activated protein kinase (MAPK) activation and attenuated apoptotic changes in cortical and hippocampal tissue [150]. More broadly, omega-3 PUFAs, particularly DHA, have been implicated in the regulation of neuroinflammation, oxidative stress, and synaptic function during brain aging [151].

In AD, attenuation of PI3K/AKT signaling and consequent activation of GSK-3β promote tau hyperphosphorylation and cognitive dysfunction [142]. In APP/PS1 mice, mTORC1 hyperactivation was linked to suppressed autophagy and impaired Aβ clearance, while human AD hippocampal tissue exhibited alterations in mTORC1 signaling and autophagy-related markers [152]. In APP/PS1 mice and human AD brain samples, dysregulation of the SIRT1/PGC-1α axis was associated with impaired mitochondrial metabolism and altered APP processing, characterized by increased β-site amyloid precursor protein cleaving enzyme 1 (BACE1) expression and reduced a disintegrin and metalloproteinase 10 (ADAM10) levels. Neuronal models further support its role in maintaining mitochondrial homeostasis and neuronal survival [153]. Collectively, these alterations converge to exacerbate tau pathology, proteostasis failure, and amyloid burden. In PD, A53T α-synuclein cellular models and human iPSC-derived dopaminergic neuron models showed that α-synuclein mutations activate the ATP citrate lyase (ACLY)-p300 pathway and suppress AMPK signaling, resulting in enhanced mTORC1 activity and autophagy inhibition. Reduced α-synuclein clearance was further demonstrated in A53T α-synuclein zebrafish and transgenic mouse models [154]. This provides a disease-specific example in which pathological α-synuclein disrupts energy-sensing and autophagic pathways, further compromising proteostatic control.

Thus, nutrient-sensing dysregulation connects metabolic disturbances with impaired autophagy, proteostasis, and inflammatory signaling through altered AKT–GSK-3β and AMPK–mTORC1 signaling. These changes gradually impair proteostasis and promote chronic inflammatory signaling. In neurodegenerative diseases, disease-related pathological mechanisms further worsen these impairments, accelerating protein accumulation and neuronal dysfunction. However, much of the mechanistic evidence is derived from cellular and animal models, and the extent to which these pathways operate similarly in human brain aging and neurodegenerative disease remains unclear. Therapeutic strategies targeting nutrient-sensing dysregulation, including mTOR inhibition, AMPK activation, oxidized nicotinamide adenine dinucleotide (NAD^+^) boosting, and modulation of insulin-related signaling, may help restore metabolic homeostasis, enhance autophagy and proteostasis, and preserve neuronal function during brain aging and neurodegeneration [155]. The main nutrient-sensing pathways involved in brain aging are shown in Figure S8.

## 11. Metabolic Drift

Metabolic drift refers to progressive alterations in the utilization, flux, and compartmentalization of major metabolic substrates during brain aging, including glucose, lipids, and mitochondrial energy metabolism. Rather than reflecting a single metabolic defect, metabolic drift involves a gradual decline in metabolic flexibility, resulting in reduced energy availability and impaired cellular adaptation. These metabolic changes occur alongside declining energy supply, mitochondrial dysfunction, oxidative stress accumulation, and lipotoxicity, resulting in neuronal dysfunction and age-related cognitive decline.

Glucose metabolism becomes less efficient in the aging brain, limiting cellular energy production. In brain microvessels isolated from C57BL/6 mice, reduced expression of 6-phosphofructo-2-kinase/fructose-2,6-bisphosphatase 3 (PFKFB3) restricts glycolytic activity through altered regulation of phosphofructokinase-1 [156]. Mitochondrial oxidative phosphorylation also declines, resulting in lower ATP production and a higher AMP/ATP ratio [12]. Consistently, aged brain microvessels exhibit reduced oxidative phosphorylation and glycolytic ATP production, accompanied by decreased glucose transporter 1 (GLUT1) and PFKFB3 expression, indicating impaired metabolic flexibility during aging [156]. These changes contribute to energetic stress, which is closely associated with mitochondrial dysfunction and progressive metabolic imbalance during brain aging.

Aging does not affect all electron transport chain complexes equally. Mitochondrial dysfunction during aging is accompanied by increased lipid peroxidation and accumulation of reactive lipid species such as 4-hydroxy-2-nonenal (4-HNE). In 4-HNE-treated rat primary cortical neurons, 4-HNE modifies mitochondrial and autophagy-related proteins, impairs mitochondrial respiration, promotes mitochondrial fragmentation, and disrupts autophagic flux [157]. These effects may limit the clearance of damaged organelles and further reinforce oxidative and metabolic stress. Lipid-derived metabolites can also affect other aspects of neuronal homeostasis. In rat cortical synaptosomes, 4-HNE modification of the glutamate transporter GLT-1, also known as excitatory amino acid transporter 2 (EAAT2), reduces glutamate uptake, increasing excitotoxic stress and synaptic dysfunction [158]. In parallel, in LPS-primed primary microglia isolated from C57BL/6 mice, ceramide-related signaling activates the NLRP3 inflammasome and promotes IL-1β secretion [159]. Thus, these experimental findings link lipid metabolic disturbance with oxidative damage, mitochondrial impairment, excitotoxicity, and inflammatory signaling, although they do not by themselves establish that the same sequence occurs during normal brain aging.

AD and PD provide disease-specific examples of how metabolic disturbances interact with pathological protein accumulation. In AD, abnormal serine phosphorylation of insulin receptor substrate 1 (IRS-1) is associated with impaired insulin signaling and increased tau phosphorylation. Abnormal IRS-1 serine phosphorylation was associated with tau pathology in human post-mortem AD and tauopathy brains, while high-fat diet-induced insulin resistance in wild-type mice increased tau phosphorylation; tau pathology in PS19 mice was also associated with IRS-1 phosphorylation [160]. Reduced insulin-degrading enzyme (IDE)-mediated clearance of Aβ has been associated with abnormal accumulation and processing of integral membrane protein 2B (BRI2), contributing to amyloid burden in post-mortem human AD hippocampus [161]. In PD, lipid peroxidation product 4-HNE promoted α-synuclein oligomerization and EV-associated propagation in primary cortical neurons and α-synuclein-expressing cellular models. In wild-type mouse models, injection of α-synuclein-containing EVs into the striatum further demonstrated the spread of α-synuclein pathology to connected brain regions, including the substantia nigra, cortex, and hippocampus [162]. In parallel, pathological α-synuclein species disrupted mitochondrial protein import through translocase of outer mitochondrial membrane 20 (TOM20)-dependent mechanisms. In SH-SY5Y and HEK293 cells, α-synuclein impaired mitochondrial protein import, whereas AAV-mediated human α-synuclein (hSNCA)-overexpressing rat models and human PD substantia nigra neurons showed corresponding mitochondrial dysfunction [163]. These mechanisms illustrate how metabolic and mitochondrial disturbances may interact with disease-associated protein pathology to further disrupt cellular homeostasis.

Taken together, metabolic drift progressively links metabolic imbalance to synaptic dysfunction and neurodegenerative progression through a continuum of energy deficit, mitochondrial dysfunction, oxidative stress, and lipotoxicity. Within this framework, mitochondrial dysfunction acts as a key connection between disrupted energy metabolism, redox imbalance, and lipid-mediated cellular damage. However, whether these metabolic alterations act as primary drivers of human brain aging or arise as secondary consequences of cellular stress and disease progression remains unclear. In neurodegenerative diseases, these metabolic alterations may further interact with disease-specific mechanisms, including impaired insulin signaling and protein clearance in AD and lipid peroxidation, α-synuclein propagation, and mitochondrial dysfunction in PD. Current evidence is largely derived from cellular and animal models, which limits the direct translation of these mechanisms to human brain aging and disease. Strategies that restore metabolic homeostasis, such as alternative energy supplementation, metabolic signaling modulation, and lifestyle interventions, may provide opportunities to improve metabolic resilience during aging and neurodegeneration, but their clinical effectiveness requires further evaluation [164]. The metabolic changes associated with brain aging and neurodegeneration are summarized in Figure S9.

## 12. Conclusions

Brain aging stems from the progressive disruption of multiple biological systems rather than from the degeneration of any single pathway. Neuroinflammation, proteostasis impairment, mitochondrial dysfunction, neurotransmitter transmission deficits, cellular senescence, gut microbiota dysbiosis, extracellular vesicle dysregulation, nutrient-sensing disturbances, and metabolic drift are closely linked. Changes in one pathway may affect others, gradually weakening neuronal homeostasis and brain function.

These interconnected pathways help explain how age-related cellular and tissue changes may increase vulnerability to neurodegenerative pathology. However, AD and PD are not simply advanced forms of normal aging; each involves disease-specific genetic, molecular, regional, and proteinopathic processes. Aβ, tau, and α-synuclein pathology may interact with aging-associated reductions in cellular resilience, thereby intensifying inflammatory, metabolic, mitochondrial, and proteostatic dysfunction.

The strength and translational relevance of the available evidence vary across the nine mechanisms discussed in this review. Neuroinflammation has comparatively broad support from both human observational or post-mortem studies and experimental models, although evidence for specific causal relationships derives mainly from experimental models. Proteostasis impairment, mitochondrial dysfunction, and neurotransmission impairment are supported by extensive experimental evidence, whereas direct evidence from the aging human brain remains more limited and largely observational. For cellular senescence and extracellular-vesicle dysregulation, mechanistic evidence derives predominantly from animal, cellular, or non-neural experimental systems, while direct human brain evidence remains limited. Gut microbiota dysbiosis and nutrient-sensing dysregulation are also supported mainly by experimental studies, but their causal roles in normal human brain aging remain insufficiently established. Evidence for metabolic drift is comparatively heterogeneous. Overall, the current mechanistic framework is supported more strongly by experimental than by human evidence, and experimental support should not be interpreted as equivalent to demonstrated causality in human brain aging.

Future studies should therefore combine longitudinal human studies, cell-type-resolved analyses, and multi-omics approaches to determine whether mechanisms identified in experimental models are recapitulated during human brain aging and to clarify their temporal and causal relationships. Potential intervention strategies targeting these aging-related mechanisms are summarized in Supplementary Table S3. Such knowledge may support the development of interventions aimed at preserving network resilience and delaying the onset of neurodegenerative disease.

## Data Availability

No new data were created or analyzed in this study. Data sharing is not applicable to this article.

## Supplementary Materials

The following supporting information can be downloaded at: https://www.mdpi.com/article/10.3390/ijms27188426/s1.

## Abbreviations

The following abbreviations are used in this manuscript:

4-HNE	4-Hydroxy-2-nonenal
β-CTF	Amyloid precursor protein β-carboxyl-terminal fragment
γH2AX	Phosphorylated histone H2AX
Aβ	Amyloid-β
AAV	Adeno-associated virus
ACLY	ATP citrate lyase
AD	Alzheimer’s disease
ADAM10	A disintegrin and metalloproteinase 10
AKT	Protein kinase B
AMP	Adenosine monophosphate
AMPK	AMP-activated protein kinase
ApoE	Apolipoprotein E
APP	Amyloid precursor protein
ASC	Apoptosis-associated speck-like protein containing a caspase recruitment domain
ASKO	α-synuclein knockout
ATG5	Autophagy-related protein 5
ATP	Adenosine triphosphate
BACE1	β-site amyloid precursor protein cleaving enzyme 1
BBB	Blood–brain barrier
BFCNs	Basal forebrain cholinergic neurons
BRI2	Integral membrane protein 2B
BSH	Bile salt hydrolase
C1q	Complement component 1q
C3	Complement component 3
CD3	Cluster of differentiation 3
CD4	Cluster of differentiation 4
CD9	Cluster of differentiation 9
CD14	Cluster of differentiation 14
CD28	Cluster of differentiation 28
CD47	Cluster of differentiation 47
CD57	Cluster of differentiation 57
CDK4/6	Cyclin-dependent kinase 4/6
cDNA	Complementary DNA
cGAS	Cyclic GMP-AMP synthase
ChAT	Choline acetyltransferase
*Clec7a*	C-type lectin domain family 7 member A
CNS	Central nervous system
CR3	Complement receptor 3
CRISPR	Clustered regularly interspaced short palindromic repeats
CSF1R	Colony-stimulating factor 1 receptor
DAMPs	Damage-associated molecular patterns
DAT	Dopamine transporter
DDR	DNA damage response
DHA	Docosahexaenoic acid
DPP4	Dipeptidyl peptidase 4
DREAM	Dimerization partner, RB-like, E2F and multi-vulval class B
DUBs	Deubiquitinases
E2F	E2F transcription factor
E18	Embryonic day 18
EAAT2	Excitatory amino acid transporter 2
EPA	Eicosapentaenoic acid
EPSP	Excitatory postsynaptic potential
ERK	Extracellular signal-regulated kinase
ERK1/2	Extracellular signal-regulated kinase 1/2
EV	Extracellular vesicle
GABA	Gamma-aminobutyric acid
GATA4	GATA binding protein 4
GLAST	Glutamate-aspartate transporter
GLT-1	Glutamate transporter 1
GLUT1	Glucose transporter 1
GSDMD	Gasdermin D
GSK-3β	Glycogen synthase kinase 3 beta
HEK293	Human embryonic kidney 293
HSC70	Heat shock cognate protein 70
hSNCA	Human α-synuclein
HSP104	Heat shock protein 104
HSP70	Heat shock protein 70
HSP90	Heat shock protein 90
HSP90α	Heat shock protein 90 alpha
HUVECs	Human umbilical vein endothelial cells
IAA	Indole-3-acetic acid
IDE	Insulin-degrading enzyme
IGF-1	Insulin-like growth factor 1
IκB	Inhibitor of κB
IKK	IκB kinase
IKK2	IκB kinase 2
IL-1α	Interleukin-1 alpha
IL-1β	Interleukin-1 beta
IL-6	Interleukin-6
IL-8	Interleukin-8
IL-18	Interleukin-18
iPLA2β	Calcium-independent phospholipase A2 beta
iPSC	Induced pluripotent stem cell
IRS-1	Insulin receptor substrate 1
K48	Lysine 48
KEAP1	Kelch-like ECH-associated protein 1
LAMP-2A	Lysosome-associated membrane protein 2A
LINE-1	Long interspersed nuclear element-1
LPS	Lipopolysaccharide
LTD	Long-term depression
LTP	Long-term potentiation
MAO-B	Monoamine oxidase B
MAPK	Mitogen-activated protein kinase
MAPKAPK2	Mitogen-activated protein kinase-activated protein kinase 2
MEFs	Mouse embryonic fibroblasts
mGluR	Metabotropic glutamate receptor
MGnD	Microglial neurodegenerative phenotype
miR-30b-5p	MicroRNA-30b-5p
MPTP	1-Methyl-4-phenyl-1,2,3,6-tetrahydropyridine
mRNA	Messenger RNA
mtDNA	Mitochondrial DNA
mTOR	Mechanistic target of rapamycin
mTORC1	Mechanistic target of rapamycin complex 1
mtROS	Mitochondrial reactive oxygen species
MVB	Multivesicular body
MyD88	Myeloid differentiation primary response 88
n-3 PUFAs	Omega-3 polyunsaturated fatty acids
NAD^+^	Oxidized nicotinamide adenine dinucleotide
NADPH	Reduced nicotinamide adenine dinucleotide phosphate
NF-κB	Nuclear factor kappa B
NGF	Nerve growth factor
NLRP3	Nucleotide-binding oligomerization domain-like receptor family pyrin domain-containing 3
NMDA	N-methyl-D-aspartate
NMDAR	N-methyl-D-aspartate receptor
NOX2	NADPH oxidase 2
NOX5	NADPH oxidase 5
NR2B	NMDA receptor subunit 2B
NR4A1	Nuclear receptor subfamily 4 group A member 1
NRF2	Nuclear factor erythroid 2-related factor 2
P2X7	P2X purinoceptor 7
p14ARF	p14 alternate reading frame
p47phox	Phagocyte oxidase 47-kDa subunit
p62	Sequestosome 1
p70S6K	p70 ribosomal S6 kinase
p75NTR	p75 neurotrophin receptor
PD	Parkinson’s disease
PET	Positron emission tomography
PFF	Preformed fibril
PFKFB3	6-Phosphofructo-2-kinase/fructose-2,6-bisphosphatase 3
PGC-1α	Peroxisome proliferator-activated receptor gamma coactivator 1-alpha
PI3K	Phosphoinositide 3-kinase
PP2A	Protein phosphatase 2A
p-p53	Phosphorylated p53
PRISMA	Preferred Reporting Items for Systematic Reviews and Meta-Analyses
pro-IL-1β	Pro-interleukin-1 beta
proNGF	Pro-nerve growth factor
PS1	Presenilin 1
RAGE	Receptor for advanced glycation end products
RB	Retinoblastoma protein
RET	Reverse electron transport
ROS	Reactive oxygen species
Rpn11	Regulatory particle non-ATPase 11
SASP	Senescence-associated secretory phenotype
SCFA	Short-chain fatty acid
SIRT1	Sirtuin 1
SIRT3	Sirtuin 3
SIRT6	Sirtuin 6
SOD2	Superoxide dismutase 2
STAT3	Signal transducer and activator of transcription 3
TβMCA	Tauro-β-muricholic acid
Tcea1	Transcription elongation factor A (SII) 1
Tert	Telomerase reverse transcriptase
TFIIS	Transcription factor IIS
TH	Tyrosine hydroxylase
TLR2	Toll-like receptor 2
TLR4	Toll-like receptor 4
TMAO	Trimethylamine N-oxide
TNF-α	Tumor necrosis factor alpha
TOM20	Translocase of outer mitochondrial membrane 20
TRAF3IP2	TRAF3-interacting protein 2
tRF-02514	Transfer RNA-derived fragment 02514
TrkA	Tropomyosin receptor kinase A
TSG101	Tumor susceptibility gene 101
ULK1	Unc-51-like kinase 1
UPS	Ubiquitin-proteasome system
VPS34	Vacuolar protein sorting 34
WT-Syn	Wild-type α-synuclein
ZO-1	Zonula occludens-1
