# Supplementary material for: Mechanisms of Brain Aging and Their Links to Alzheimer’s and Parkinson’s Disease Pathology
Source: Int J Mol Sci. 2026 Sep 21;27(18):8426. doi: 10.3390/ijms27188426 (PMC13607847; doi:10.3390/ijms27188426)
Supplement: Supplementary file 1 [file ijms-27-08426-s001.zip › ijms-4504710-supplementary figures.pdf]

Supplementary Figure

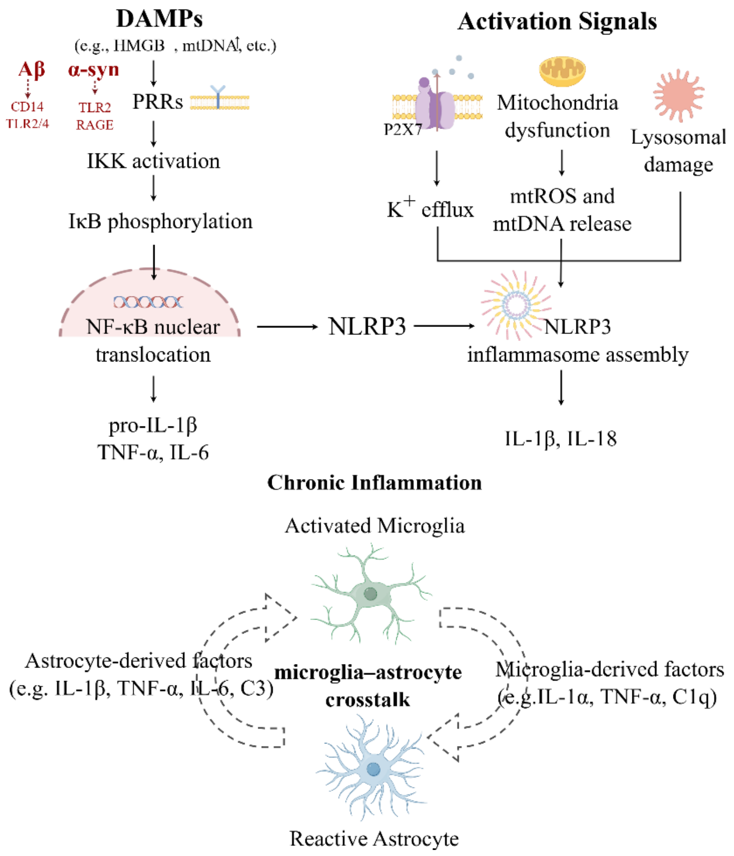

Figure S1. Neuroinflammatory pathways during brain aging. Age-associated DAMPs, together with AD- and PD-related factors such as Aβ and α-synuclein, activate inflammatory signaling involving NF-κB, NLRP3, and microglia–astrocyte interactions. These processes promote cytokine production and sustained neuroinflammation. Solid arrows indicate experimentally supported relationships, whereas dashed arrows indicate proposed, context-dependent, or cross-model relationships. Created using Figdraw.com (License ID: YTIPI33c30).

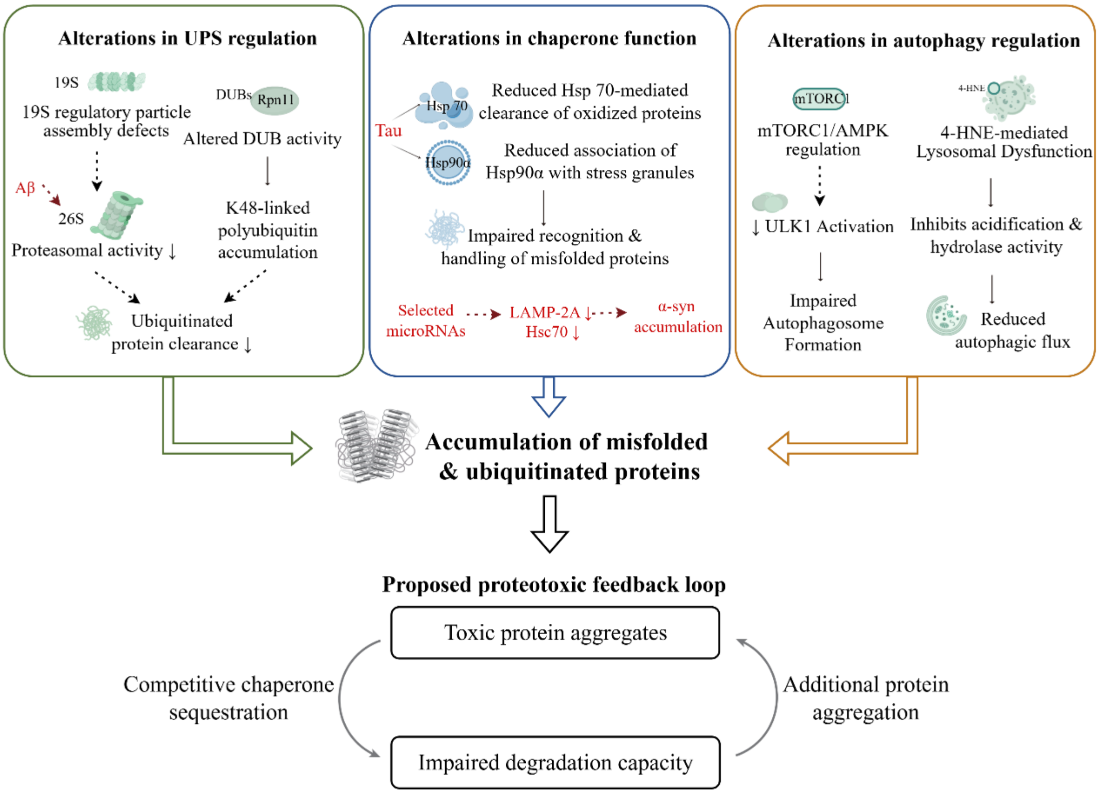

Figure S2. Proteostasis impairment during brain aging and neurodegeneration. Aging-related disruption of the UPS, molecular chaperones, and autophagy–lysosomal pathways reduce the clearance of damaged and misfolded proteins. Accumulation of Aβ, tau, and α-synuclein can further impair protein-quality-control systems and promote proteotoxic stress. The schematic integrates evidence from multiple cellular, animal, and human studies. Solid arrows indicate experimentally supported relationships, whereas dashed arrows indicate proposed, context-dependent, or cross-model relationships. Created using Figdraw.com (License ID: SWUOP92290).

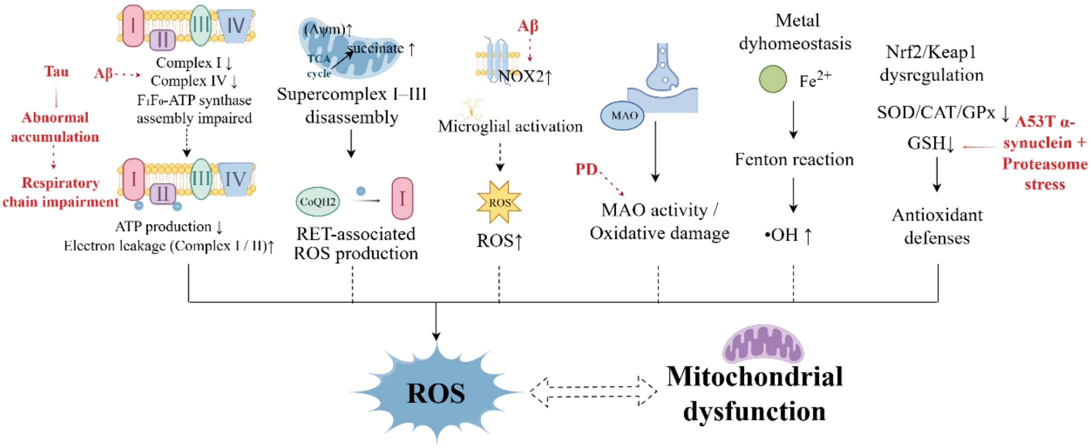

Figure S3. Oxidative stress and mitochondrial dysfunction during brain aging and neurodegeneration. Multiple sources of ROS, including respiratory-chain impairment, RET, NOX2 activation, monoamine oxidase activity, metal dyshomeostasis, and reduced antioxidant defenses, contribute to oxidative stress and mitochondrial dysfunction. AD- and PD-related factors, including Aβ, tau, and α-synuclein, can further influence these pathways in disease-specific experimental contexts. The schematic integrates evidence from multiple experimental systems. Solid arrows indicate experimentally supported relationships, whereas dashed arrows indicate proposed, context-dependent, or cross-model relationships. Created using Figdraw.com (License ID: RSPOS74bb4).

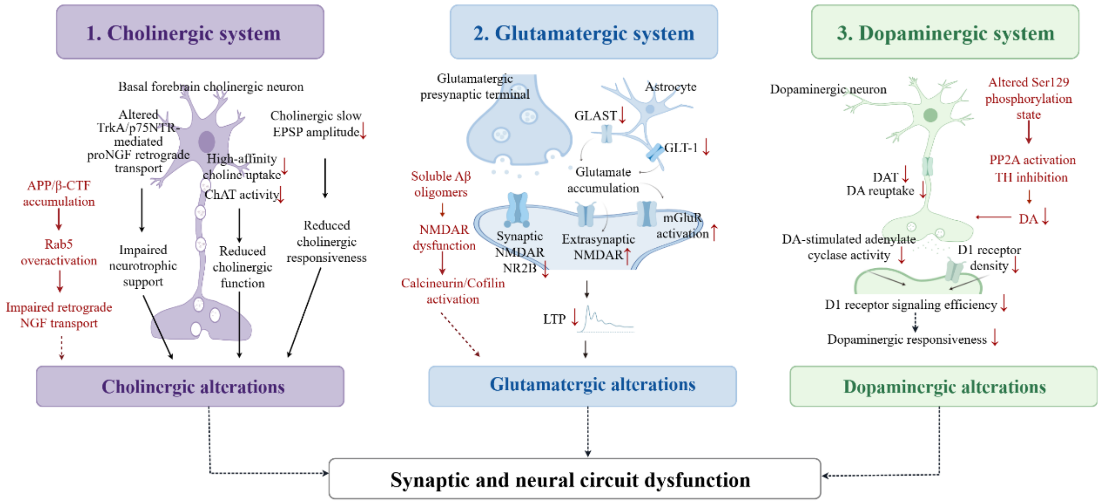

Figure S4. Neurotransmission impairment during brain aging and neurodegeneration. Age-related alterations in cholinergic, glutamatergic, and dopaminergic signaling disrupt neurotransmitter synthesis, transport, receptor function, and synaptic plasticity, thereby impairing neural-circuit regulation. AD- and PD-related factors, including APP/β-CTF, Aβ, and α-synuclein, can further aggravate neurotransmission deficits in disease-specific contexts. The schematic integrates evidence from multiple experimental systems. Solid arrows indicate experimentally supported relationships, whereas dashed arrows indicate proposed, context-dependent, or cross-model relationships. Created using Figdraw.com (License ID: SSOWW3a3a3).

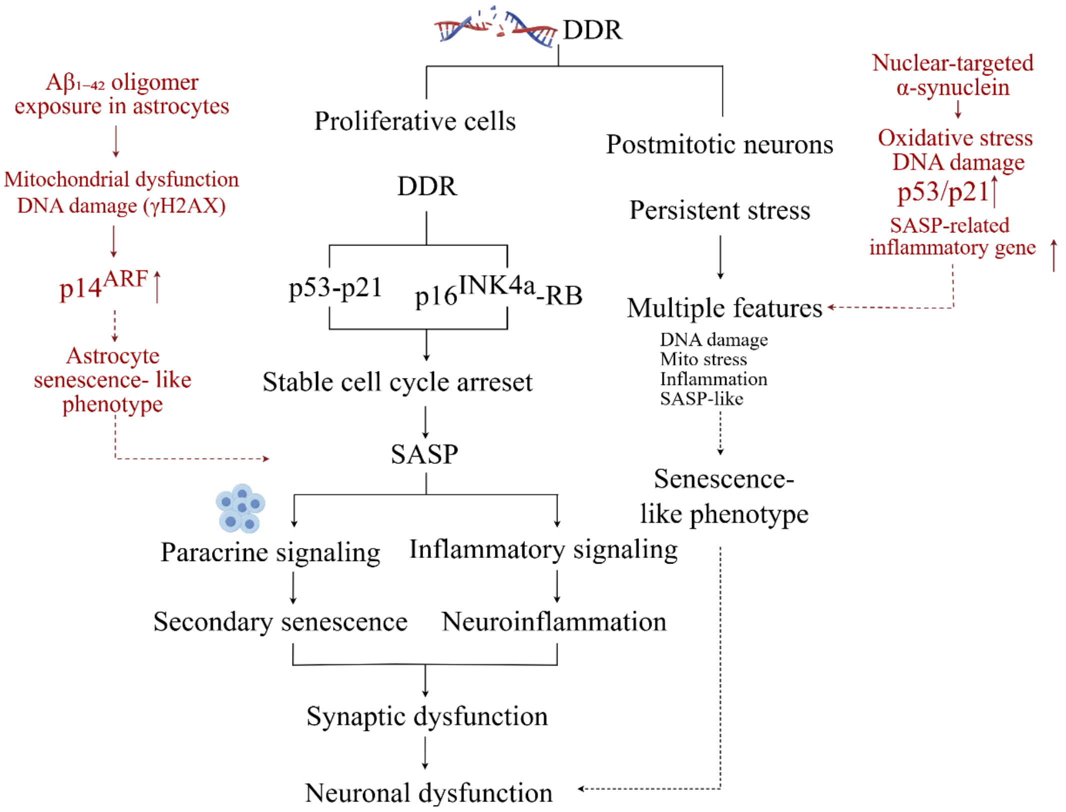

Figure S5. Cellular senescence-related signaling during brain aging. Persistent DNA damage activates p53–p21 and p16^INK4a–RB pathways in proliferative cells, promoting stable cell-cycle arrest and SASP signaling. In postmitotic neurons, senescence-like states are characterized by multiple stress-related features, including DNA damage, mitochondrial dysfunction, inflammation, and SASP-like changes, rather than proliferative arrest. Astrocyte- and α-synuclein-related pathways further illustrate cell type- and disease-specific senescence-associated responses. SASP-mediated paracrine and inflammatory signaling can promote secondary senescence, neuroinflammation, and synaptic dysfunction. The schematic integrates evidence from multiple experimental systems. Solid arrows indicate experimentally supported relationships, whereas dashed arrows indicate proposed, context-dependent, or cross-model relationships. Created using Figdraw.com (License ID: RIPYWd902e)

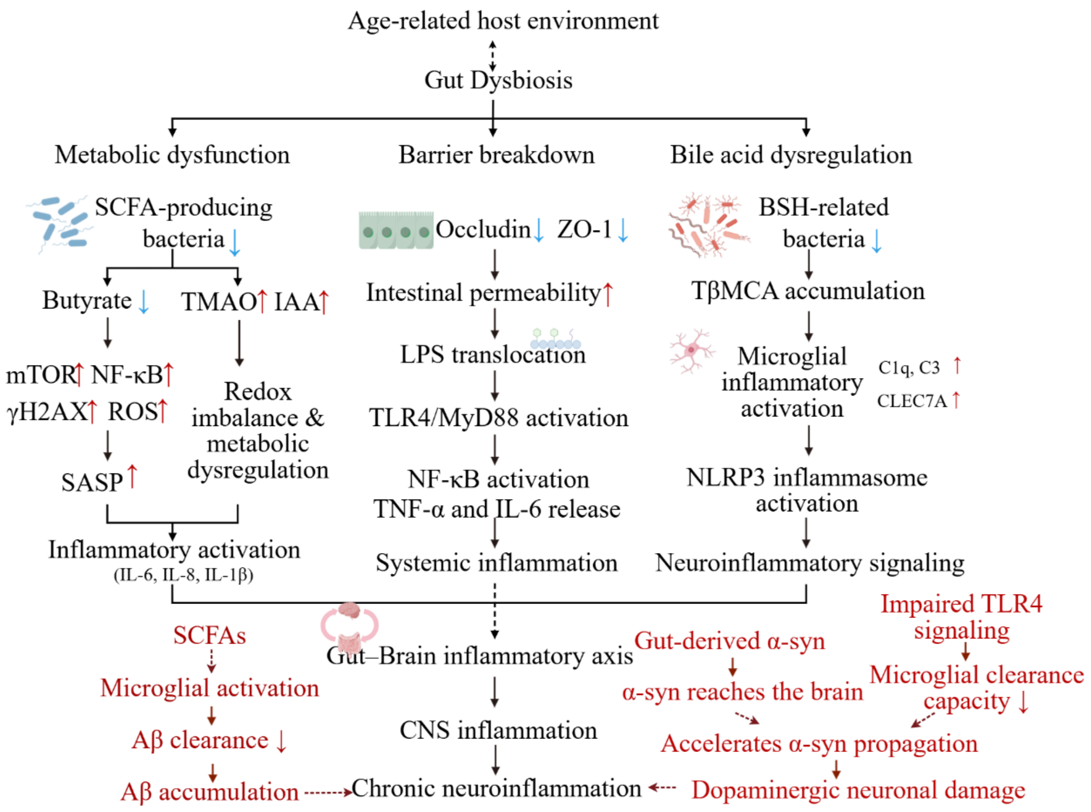

Figure S6. Gut microbiota dysbiosis during brain aging and neurodegeneration. Age-related alterations in microbial metabolites, intestinal barrier integrity, and bile-acid metabolism can promote systemic and central inflammatory signaling. Reduced short-chain fatty acid production, increased intestinal permeability, and bile-acid dysregulation are linked to microglial activation and neuroinflammation. In AD-related contexts, impaired microglial clearance may favor Aβ accumulation, whereas in PD-related contexts, gut-derived α-synuclein and impaired TLR4-dependent clearance may promote α-synuclein propagation and dopaminergic neuronal damage. The gut microbiota–host relationship is bidirectional: age-related changes in the host metabolic and immune environment may also contribute to microbial remodelling. The schematic integrates evidence from multiple experimental systems. Solid arrows indicate experimentally supported relationships, whereas dashed arrows indicate proposed, context-dependent, or cross-model relationships. Created using Figdraw.com (License ID: OOATYd8548).

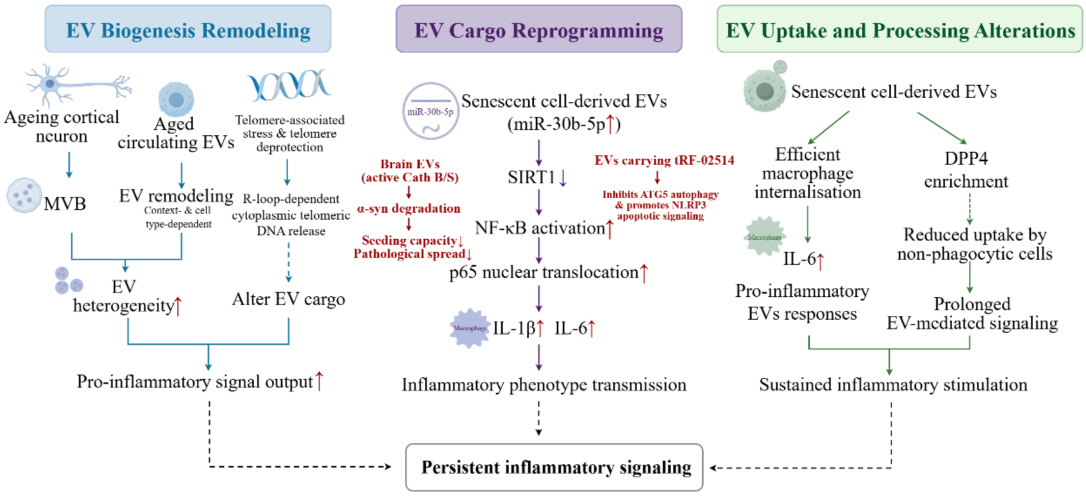

Figure S7. Extracellular-vesicle dysregulation during brain aging and neurodegeneration. Aging and cellular senescence alter extracellular-vesicle (EV) biogenesis, cargo composition, cellular uptake, and persistence. Senescence-associated EV cargo, including miR-30b-5p, can suppress SIRT1 and enhance NF-κB-dependent inflammatory signaling in recipient cells, while altered EV uptake may prolong intercellular signaling. In AD- and PD-related contexts, EVs can influence pathological protein transport, inflammatory signaling, and protein clearance in a source- and cargo-dependent manner. The schematic integrates evidence from multiple experimental systems. Solid arrows indicate experimentally supported relationships, whereas dashed arrows indicate proposed, context-dependent, or cross-model relationships. Created using Figdraw.com (License ID: IORYYa9ba2).

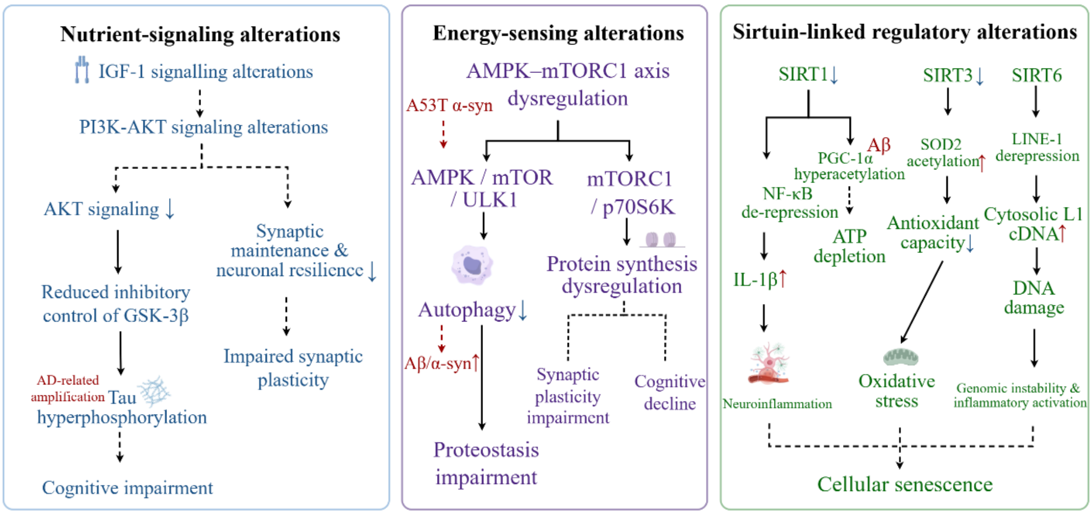

Figure S8. Nutrient-sensing dysregulation during brain aging and neurodegeneration. Altered IGF-1/PI3K–AKT, AMPK–mTORC1, and sirtuin signaling disrupts synaptic plasticity, proteostasis, antioxidant defense, and cellular homeostasis. In AD-related contexts, impaired PI3K–AKT signaling is linked to tau hyperphosphorylation, whereas in PD-related models, α-synuclein can suppress AMPK signaling and impair autophagic clearance. The schematic integrates findings from different cellular, animal, and human studies and does not imply that all relationships were demonstrated within a single experimental model. Solid arrows indicate experimentally supported relationships, whereas dashed arrows indicate proposed, context-dependent, or cross-model relationships. Created using Figdraw.com (License ID: USWAI88f88).

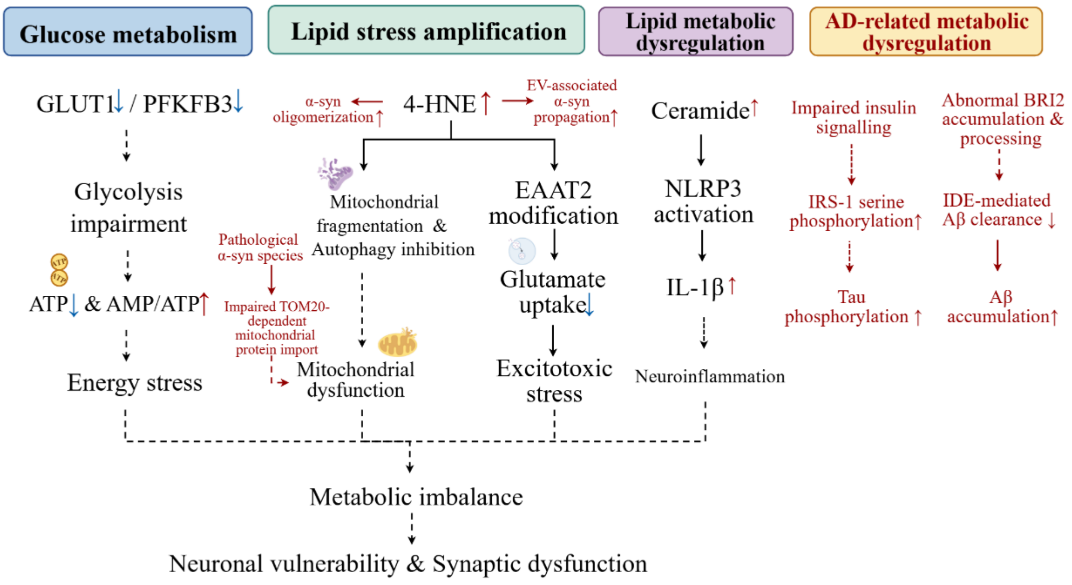

Figure S9. Metabolic drift during brain aging and neurodegeneration. Impaired glucose metabolism, lipid stress, and mitochondrial dysfunction contribute to energy stress, neuroinflammation, excitotoxicity, and synaptic vulnerability. In AD-related contexts, insulin resistance and impaired Aβ clearance are associated with metabolic dysfunction, whereas in PD-related models, α-synuclein and 4-HNE can disrupt mitochondrial homeostasis. The schematic integrates findings from different cellular, animal, and human studies and does not imply that all relationships were demonstrated within a single experimental model. Solid arrows indicate experimentally supported relationships, whereas dashed arrows indicate proposed, context-dependent, or cross-model relationships. Created using Figdraw.com (License ID: ASTUAee50b).
